# Histone demethylase JMJD1A coordinates acute and chronic adaptation to cold stress via thermogenic phospho-switch

**DOI:** 10.1038/s41467-018-03868-8

**Published:** 2018-04-19

**Authors:** Yohei Abe, Yosuke Fujiwara, Hiroki Takahashi, Yoshihiro Matsumura, Tomonobu Sawada, Shuying Jiang, Ryo Nakaki, Aoi Uchida, Noriko Nagao, Makoto Naito, Shingo Kajimura, Hiroshi Kimura, Timothy F. Osborne, Hiroyuki Aburatani, Tatsuhiko Kodama, Takeshi Inagaki, Juro Sakai

**Affiliations:** 1 Division of Metabolic Medicine, Research Center for Advanced Science and Technology, The University of Tokyo, Tokyo, 153-8904 Japan; 2 Niigata College of Medical Technology, Niigata, 950-2076 Japan; 3 Rhelixa Inc., Tokyo, 101-0032 Japan; 4 Genome Science Division, Research Center for Advanced Science and Technology, The University of Tokyo, Tokyo, 153-8904 Japan; 5 Department of Pathology, Niigata Medical Center, Niigata, 950-2022 Japan; 6 Department of Cell and Tissue Biology, UCSF Diabetes Center, University of California, San Francisco, San Francisco, CA 94143-0669 USA; 7 Cell Biology Unit, Institute of Innovative Research, Tokyo Institute of Technology, Yokohama, 226-8503 Japan; 8 Metabolic Disease Program, Sanford-Burnham Medical Research Institute, Orlando, FL 32827 USA; 9 Laboratory for Systems Biology and Medicine, Research Center for Advanced Science and Technology, The University of Tokyo, Tokyo, 153-8904 Japan; 10 Laboratory of Epigenetics and Metabolism, Institute for Molecular and Cellular Regulation, Gunma University, Gunma, 371-8512 Japan; 11 Division of Molecular Physiology and Metabolism, Tohoku University Graduate School of Medicine, Sendai, 980-8574 Japan

## Abstract

In acute cold stress in mammals, JMJD1A, a histone H3 lysine 9 (H3K9) demethylase, upregulates thermogenic gene expressions through β-adrenergic signaling in brown adipose tissue (BAT). Aside BAT-driven thermogenesis, mammals have another mechanism to cope with long-term cold stress by inducing the browning of the subcutaneous white adipose tissue (scWAT). Here, we show that this occurs through a two-step process that requires both β-adrenergic-dependent phosphorylation of S265 and demethylation of H3K9me2 by JMJD1A. The histone demethylation-independent acute *Ucp1* induction in BAT and demethylation-dependent chronic *Ucp1* expression in beige scWAT provides complementary molecular mechanisms to ensure an ordered transition between acute and chronic adaptation to cold stress. JMJD1A mediates two major signaling pathways, namely, β-adrenergic receptor and peroxisome proliferator-activated receptor-γ (PPARγ) activation, via PRDM16-PPARγ-P-JMJD1A complex for beige adipogenesis. S265 phosphorylation of JMJD1A, and the following demethylation of H3K9me2 might prove to be a novel molecular target for the treatment of metabolic disorders, via promoting beige adipogenesis.

## Abstract (web summary)

JMJD1A is essential for thermogenic gene induction in brown adipose tissue. Here the authors show that white adipose tissue beige-ing requires both β-adrenergic-dependent phosphorylation of S265 and demethylation activity of JMJD1A while brown adipose tissue-driven thermogenesis requires β-adrenergic dependent phosphorylation of S265 but is independent of H3K9me2 demethylation.

## Introduction

Cold stress is a major threat for warm-blooded animals, and therefore adaptive thermogenesis to combat cold stress is crucial for survival. Brown adipose tissue (BAT) specifically plays a critical and a rapid role in thermogenesis by dissipating chemical energy to produce heat (i.e., nonshivering thermogenesis). Recent studies indicate that mammals have an additional type of thermogenic adipocyte, termed beige (or brite), that resides in predominantly subcutaneous white adipose depots that provides a more sustained thermogenic response to chronic cold stress (ref. ^1^, also reviewed in refs. ^2,3^). The signature thermogenic protein in both brown and beige adipocytes is uncoupling protein-1 (UCP1), which stimulates thermogenesis by uncoupling cellular respiration from mitochondrial ATP synthesis^4,5^; however, BAT and beige cells display different temporal patterns of *Ucp1* expression that likely contribute to their different roles in the comprehensive thermogenic response^2^. In BAT, *Ucp1* is expressed at constitutively high basal levels before cold stress^6^, whereas in subcutaneous white adipose tissue (scWAT), *Ucp1* expression is normally very low, but it is induced to a very high level in response to chronic environmental cold stress (and also in response to the exposure to agonists for β-adrenergic receptor (BAR) or peroxisome proliferator-activated receptor-γ (PPARγ)^1,7^). The actions of *Ucp1*-dependent thermogenesis in BAT and scWAT beige cells likely provide a coordinated adaptive response to efficiently link the acute and chronic phases of cold stress-dependent uncoupled respiration.

JMJD1A (also known as JHDM2A or KDM3A) is an histone H3 lysine 9 (H3K9) dimethyl and monomethyl (me2/1) demethylase, with broad functional roles in stem cell renewal^8^, spermatogenesis^9^, sex determination^10^, and cancer^11,12^. It is also a crucial regulator of normal body weight control and acute thermogenesis in BAT^13–15^. A previous study reported that BAR-dependent induction of *Ucp1* in brown adipocytes under acute cold exposure required JMJD1A demethylase^14^. They proposed that the activation of *Ucp1* required JMJD1A demethylase activity, because BAR stimulation in a cultured line of brown adipocytes resulted in a decrease of H3K9me2 on the *Ucp1* gene enhancer, and this effect was lost when JMJD1A levels were reduced by a knockdown approach. This result was unexpected because *Ucp1* is constitutively expressed at a high basal level in BAT before cold stress, indicating that the *Ucp1* locus already has features of euchromatin. Therefore, a high level of the repressive H3K9me2 histone mark at the *Ucp1* locus in BAT was not expected. In addition, alterations in histone methylation are usually associated with long-term lasting memory transitions, such as terminal cell differentiation (reviewed in refs. ^16–18^). Therefore, the previous study was potentially complicated, and more detailed studies were necessary to address the functional role of JMJD1A in thermogenic regulation.

In a more recent report, we uncovered a novel role for JMJD1A in acute induction of *Ucp1* and *Adrb1* gene expression in BAT in response to BAR signaling. We showed that BAR signaling resulted in PKA-dependent phosphorylation and chromatin recruitment of JMJD1A to PPARγ target sites, in *Ucp1* and *Adrb1* locus regulatory regions^13^. Importantly, the induction was not accompanied by a decrease in H3K9me2 of the *Ucp1* enhancer (Supplementary Fig. 3b in ref. ^13^), nor did it depend on JMJD1A demethylase activity (Fig. 3d in ref. ^13^). Instead, we showed that after phosphorylation, JMJD1A facilitated long-range chromatin interactions to facilitate BAR signal-dependent gene expression (i.e., through dynamic higher ordered chromatin structure)^13^. This phosphorylation-dependent, but H3K9me2 demethylation-independent BAR induction mechanism functions on top of the other chromatin regulatory events (e.g., histone acetylation^13^) that allow constitutive high expression of *Ucp1* in BAT.

Acute cold stress triggers phosphorylation cascades leading to immediate heat production in BAT, while chronic cold stress promotes a lasting adaptive thermogenic phenotype through activation of the beige cell program in scWAT that is thought to be associated with the alterations in methylation of DNA and/or histones^19^. However, how chronic cold stress and downstream β-adrenergic signaling is sensed by epigenetic enzymes, and how they provide a sustained thermogenic response was not understood. Therefore, we investigated a putative role for BAR-activated JMJD1A in beigeing of scWAT.

## Results

### H3K9me2-independent thermogenic gene inductions in BAT

**Fig. 1:**
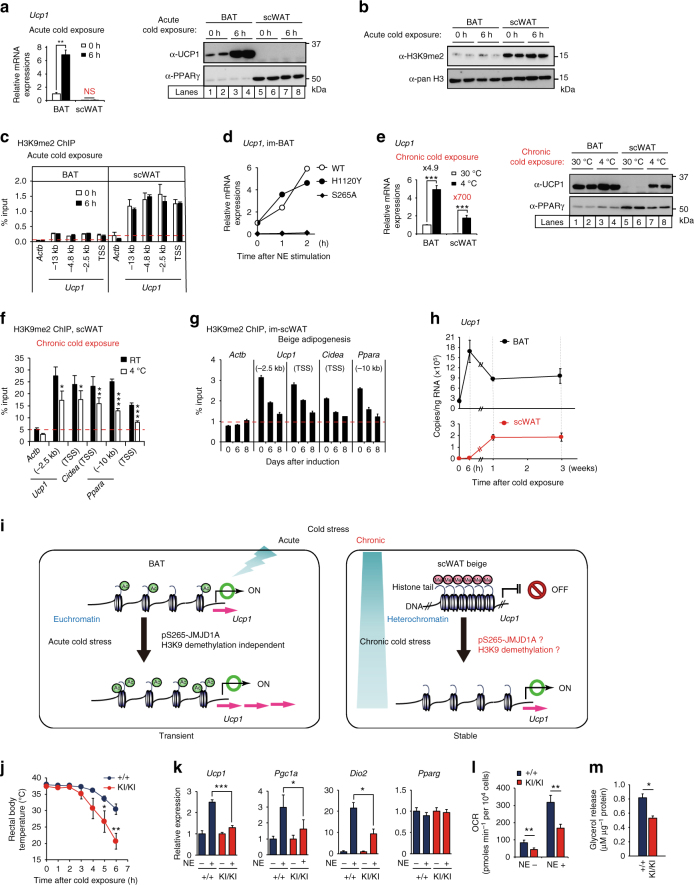
*Ucp1* expression and H3K9me2 levels in adipose tissues and BAT thermogenic function in *Jmjd1a*-S265A^KI/KI^ mice . **a** *Ucp1* mRNA (*n* = 3) (left) and protein levels detected by immunoblotting (IB) (right) in BAT, and scWAT of mice exposed to 4 °C for 6 h. **b** H3K9me2 immunoblotting using purified histones from adipose tissues of mice housed at RT. **c** H3K9me2 ChIP-qPCR in BAT, and scWAT of mice exposed to 4 °C for 6 h (*n* = 3). **d** NE-induced *Ucp1* mRNA levels in im-BATs stably expressing WT or indicated mutants of hJMJD1A. The relative quantity in im-BATs expressing WT-JMJD1A on day 8 before NE treatment (0 h) is defined as 1. **e** *Ucp1* mRNA expressions (*n* = 3) (left) and proteins (right) in BAT, and scWAT from mice exposed to 30 °C or 4 °C for 1 week. Uncropped images of the blots (**a**, **b**, **e**) are shown in Supplementary Fig. 8. **f**,** g** H3K9me2 ChIP-qPCR in scWAT from mice placed at 4 °C for 1 week (*n* = 4) (**f**) and in im-scWATs during beige adipogenesis (mean ± s.e.m. of three technical replicates) (**g**). **h** Bi-phasic *Ucp1* expressions during cold exposure in BAT and scWAT. Mice were exposed to 4 °C for the indicated time, and *Ucp1* mRNAs in BAT and scWATs were quantified by qPCR (*n* = 3). The data were converted to copy number per ng of total RNA. **i** In BAT, *Ucp*1 locus is in euchromatin (left), while in scWAT, it is in heterochromatin with H3K9me2 (right). In BAT, cold exposure leads to acute induction of *Ucp1* mRNA through the mechanisms independent of H3K9me2 demethylation (left). In scWAT, H3K9me2 at *Ucp1* gene locus needs to be removed for beige adipogenesis (right bottom). **j** Cold intolerance in *Jmjd1a*-S265A^KI/KI^ mice (*n* = 6 per genotype group). Shown is the body temperature of 9-week-old mice at different times after cold exposure (4 °C). **k** Impaired NE-induced activation of selective genes in BAT (*n* = 6 per genotype group). **l**, **m** Reduced NE-induced mitochondrial respiration (**l**) and NE-induced glycerol release (**m**) in primary brown adipocytes from *Jmjd1a*-S265A^KI/KI^ mice. Data are mean ± s.e.m. of five technical replicates (**l**) and three independent experiments (**m**). Data are mean ± s.e.m. **a**,** c**, **e**, **f**, **j**, **k** Student’s *t *test (**a**, **e**, **f**, **j**, **l**, **m**) or analysis of variance, followed by Tukey’s post hoc comparison (**k**) were performed for comparisons. *P < 0.05, **P < 0.01, and ***P < 0.005

H3K9me2 is a signature heterochromatin mark that facilitates chromatin condensation and gene silencing^20^. *Ucp1* is expressed at high constitutive levels in BAT, but at very low levels in scWAT. The high constitutive expression in BAT is consistent with the significant association of the gene enhancer and promoter activation histone marks H3K27ac, and H3K4me3 with the *Ucp1* in BAT chromatin (Supplementary Fig. 1a). This is consistent with the selective activation of *Ucp1* after the cold exposure in BAT (Fig. 1a). This is also consistent with the significantly lower levels of total H3K9me2 in histones (Fig. 1b) and lower levels of H3K9me2 associated with *Ucp1* enhancer/promoter regions in BAT, relative to scWAT (Fig. 1c). Consistent with our previous studies, the acute activation of *Ucp1* was dependent on PKA phosphorylation of JMJD1A, because the S265A mutant failed to induce *Ucp1* expression (Fig. 1d). However, acute activation of *Ucp1* in BAT was independent of JMJD1A demethylase activity, because a mutation of the catalytic histidine to tyrosine (H1120Y) that eliminates demethylase activity^13^ had no effect (Fig. 1d).

### H3K9me2-dependent beige-selective gene inductions in scWAT

In addition to the rapid activation of thermogenesis in BAT by cold stress, chronic exposure of mice to cold results in markedly induced *Ucp1* mRNA and proteins in scWAT, referred to as beigeing (reviewed in refs. ^2,3,21^) (Fig. 1e and Supplementary Fig. 1b). Given that *Ucp1* enhancer/promoter regions are heavily H3K9me2 methylated and silenced in scWAT before and during acute cold exposure, we reasoned that the activation of the beigeing-thermogenic program during chronic cold exposure might be associated with H3K9me2 demethylation of thermogenic gene enhancer/promoter regions. This would be consistent with prior studies, demonstrating that long-term stable transcriptional changes are associated with altered chromatin methylation^20^. To examine this hypothesis, we measured the enhancer/promoter region-associated H3K9me2 levels by chromatin immunoprecipitation (ChIP) analysis, using scWAT of the mice housed at room temperature (RT) vs. at 4 °C for 1 week. This analysis revealed that H3K9me2 on the *Ucp1*, *Cidea*, and *Ppara* gene enhancer/promoter regions in scWAT was significantly reduced following chronic cold exposure of mice (Fig. 1f), while H3K9me2 in BAT, which is very low even before cold exposure (see also Fig. 1c), were not significantly decreased after 1 week of cold exposure (Supplementary Fig. 1c). This chronic cold exposure also resulted in elevated *Ucp1* expression in scWAT (Fig. 1e and Supplementary Fig. 1b). We also observed a time-dependent decrease in *Ucp1* gene-associated H3K9me2 during in vitro differentiation of beige adipocytes, derived from scWAT cultured with rosiglitazone (ROS) (Fig. 1g). This was also true for other key beige adipocyte genes (Fig. 1g). ROS also enhanced the norepinephrine (NE)-dependent increase in *Ucp1* expression, and oxygen consumption observed the following scWAT differentiation in vitro (Supplementary Fig. 1d–g).

To directly compare the acute activation of *Ucp1* in BAT vs. the chronic activation in scWAT, we monitored *Ucp1* over the course of a 3-week exposure of mice to 4 °C. *Ucp1* mRNA in BAT was acutely activated and peaked early (6 h), and it declined over the course of 1 week to half the peak value. In scWAT, *Ucp1* mRNA was barely detected during the acute phase of cold exposure; however, it significantly increased over the first week and remained at this elevated steady-state level over the 3-week exposure period (Fig. 1h). The long-term increase in *Ucp1* expression in scWAT is also associated with a decrease in H3K9me2, at the *Ucp1* locus in scWAT, during chronic cold exposure (Fig. 1f). These different responses raised the hypothesis that while pS265-JMJD1A acutely induces thermogenic gene expressions in BAT through a mechanism that is independent of H3K9me2 demethylation activity, in scWAT, pS265-JMJD1A might also erase H3K9me2 to provide long-term activation of thermogenic genes required for adaptation to chronic cold exposure (Fig. 1i).

### Generation of *Jmjd1a*-S265A^KI/KI^ mice

To study the physiological relevance of pS265-JMJD1A as a cold sensor in BAT, and also the relationship with the H3K9me2 changes in scWAT beigeing (Fig. 1i), we generated mice with a serine 265-to-alanine knock-in mutation (*Jmjd1a*-S265A^KI/KI^). *Jmjd1a*-S265A^KI/KI^ mice were born in normal Mendelian ratio and did not exhibit male infertility or male-to-female sex reversal, observed in *Jmjd1a*-null mice^9,10^ (Supplementary Fig. 1h–m). When fed chow and housed at RT, *Jmjd1a*-S265A^KI/KI^ mice had slightly elevated body weights, relative to wild-type (*WT*) mice (Supplementary Fig. 1n). However, similar to *Jmjd1a*-null mice^13,14^, *Jmjd1a*-S265A^KI/KI^ mice had reduced BAT thermogenic activity: the whole animal oxygen consumption, following injection of NE in mice acclimated to 30 °C^4,22,23^, was 20% lower in *Jmjd1a*-S265A^KI/KI^ mice, consistent with reduced BAT function (Supplementary Fig. 1o), and exhibited defective adaptive thermogenesis in response to acute cold exposure (Fig. 1j) and a blunted NE-induced upregulation of three key genes (*Ucp1*, *Pgc1a*, and *Dio2*) involved in thermogenesis in BAT (Fig. 1k). Additionally, NE-stimulated oxygen consumption and glycerol release were profoundly reduced in primary brown adipocytes from *Jmjd1a*-S265A^KI/KI^ mice as well (Fig. 1l, m). In adulthood, like *Jmjd1a*-null mice, *Jmjd1a*-S265A^KI/KI^ mice also had features of altered BAT, including larger depot size and increased fat accumulation (Supplementary Fig. 1p, q).

### S265 phosphorylation of JMJD1A induces beige-selective genes

**Fig. 2:**
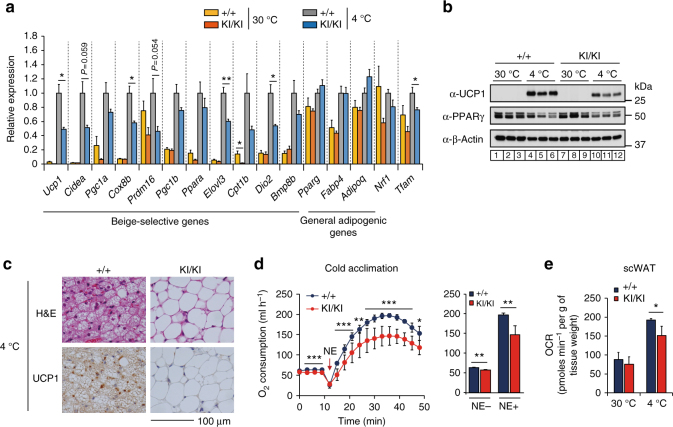
Phospho-S265 JMJD1A induces beige biogenesis. **a** qPCR analysis demonstrates decreased expression of beige-selective genes in scWAT from *Jmjd1a*-S265A^KI/KI^ mice exposed to 30 °C or 4 °C for 1 week (*n* = 5 per genotype group). **b** Immunoblot analysis of UCP1 and PPARγ in tissue homogenates of scWAT from mice presented in **a**. Uncropped images of the blots are shown in Supplementary Fig. 8. **c** Haematoxylin and eosin (H&E) and UCP1 staining sections of scWAT from *WT* and *Jmjd1a*-S265A^KI/KI^ mice exposed to chronic cold exposure (4 °C for 1 week) (scale bar, 100 μm). **d** NE-induced oxygen consumption rate (OCR) in mice exposed to chronic cold exposure (4 °C for 4 weeks) (*n* = 7 per genotype group) (left). OCR before and 30 min after NE treatment are analyzed (right) (*n* = 7). **e** OCR of scWAT from mice exposed to 30 °C or 4 °C for 1 week (*WT*: *n* = 3; *Jmjd1a*-S265A^KI/KI^: *n* = 4). Data are mean ± s.e.m. **a**, **d**, **e** Analysis of variance were performed followed by Tukey’s post hoc comparison in **a**. Student’s *t *test was performed for comparisons in **d**,** e**. *P < 0.05, **P < 0.01, and ***P < 0.005 were considered statistically significant

To study whether the pS265-JMJD1A was involved in the induction of beige-selective genes, we quantified the expression of thermogenic genes in the scWAT of *WT* and *Jmjd1a-*S265A^KI/KI^ mice housed at 30 °C or following cold temperature (4 °C) for 1 week. A 4 °C treatment induced the expression of the core set of thermogenic genes in scWAT, including *Ucp1*, *Cidea*, *Pgc1a*, *Cox8b*, *Elovl3*, and *Dio2* in *WT* mice (Fig. 2a), but this response was severely attenuated in the scWAT of *Jmjd1a*-S265A^KI/KI^ mice. By contrast, expression of general adipogenic genes (*Pparg* and *Adipoq*) did not differ significantly between *WT* and *Jmjd1a-*S265A^KI/KI^ mice. Consistent with the mRNA levels, UCP1 protein levels increased after cold exposure in the scWAT of the *WT* mice, while they increased by only about half as much in *Jmjd1a-*S265A^KI/KI^ mice (Fig. 2b). Histological staining showed a lack of UCP1-positive beige adipocytes with multilocular lipid droplets in *Jmjd1a-*S265A^KI/KI^ mice after cold exposure (Fig. 2c). Basal whole-body oxygen consumption rate (OCR) before NE injection was significantly higher in the 4 °C-acclimated *WT* mice (55 ml h^−1^; Fig. 2d), than in the 30 °C-acclimated *WT* mice (40 ml h^−1^; Supplementary Fig. 1o). However, OCR response was 20–25% lower in *Jmjd1a*-S265A^KI/KI^ mice, relative to *WT* mice (Fig. 2e). This suggests that S265 phosphorylation of JMJD1A is important in beigeing, as it is in the acute thermogenic response in BAT. Additionally, the abundant clusters of UCP1-positive adipocytes with multilocular lipid droplets and UCP1, that develop in scWAT from inguinal fat pads of *WT* mice following chronic cold exposure, were significantly reduced in both *Jmjd1a*-S265A^KI/KI^ and *Jmjd1a*-null mice (Supplementary Fig. 2).

### pS265-JMJD1A cell autonomously induces beige-selective genes

**Fig. 3:**
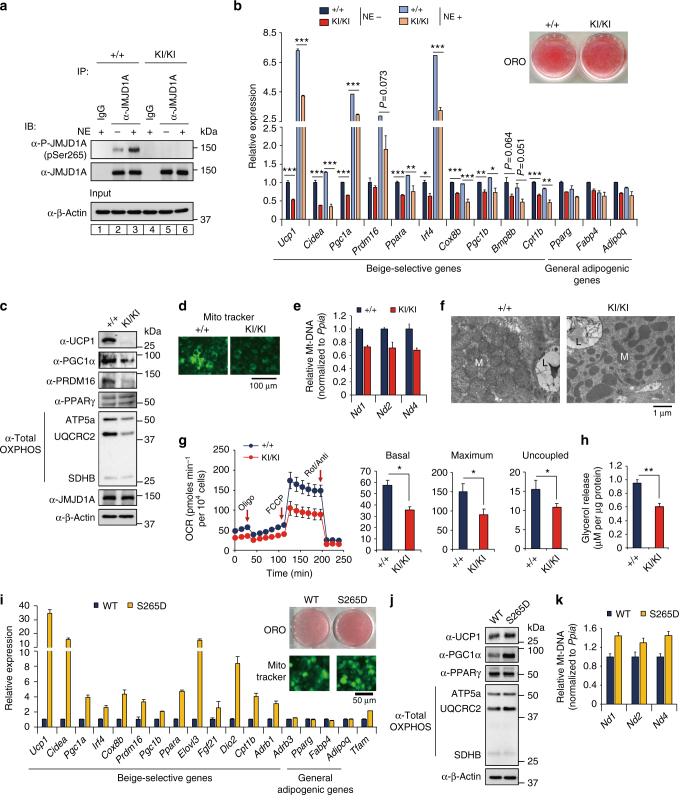
P-JMJD1A cell autonomously induces beige adipogenesis. **a** pSer265-JMJD1A protein levels in *WT* (+/+) and S265A knock-in whole-cell lysates (WCL) from scWAT cultures treated with NE or vehicle for 1 h. **b** Decreased beige-selective gene expressions in S265A knock-in scWAT cultures treated with NE (10 μM) for 2 h (mean ± s.e.m. of three technical replicates). ORO staining of indicated genotype of scWAT cultures (inset). **c** Immunoblot analysis using anti-UCP1, anti-PGC1α, anti-PRDM16, anti-PPARγ, or anti-total OXPHOS antibodies cocktail, using WCL from WT and S265A knock-in scWAT cultures. **d** MitoTracker staining in indicated genotype scWAT cultures (scale bar, 100 μm). **e** Mitochondrial DNA (mt-DNA) contents measured by qPCR in indicated scWAT cultures (mean ± s.e.m. of three independent experiments). **f** Electron micrographs of indicated genotype of scWAT cultures (bar, 1 μm). Mitochondria (M) and lipid droplets (L) are indicated. **g** The OCR of indicated scWAT cultures (left). The arrows indicate the time of addition for oligomycin (Oligo), FCCP, and rotenone/antimycin A (Rot/Anti). Basal, maximum, and uncoupled respiration were calculated (mean ± s.e.m. of five technical replicates) (right). **h** Glycerol release from indicated scWAT cultures after the treatment with NE for 3 h (mean ± s.e.m. of three independent experiments). **i** Increased expressions of beige-selective genes in S265D-hJMJD1A-transduced im-scWATs (mean ± s.e.m. of three technical replicates). ORO staining and MitoTracker staining (inset) (scale bar, 50 μm). **j** Immunoblotting with anti-UCP1, anti-PGC1α, anti-PPARγ, or anti-total OXPHOS antibodies cocktail using WCL from indicated im-scWATs. Uncropped images of the blots (**a**, **c**, **j**) are shown in Supplementary Fig. 8. **k** Mitochondrial DNA content measured by qPCR in indicated im-scWATs (mean ± s.e.m. of three technical replicates). Student’s *t* test was performed for comparisons in **b**, **g**, **h**. *P < 0.05, **P < 0.01, and ***P < 0.005 were considered statistically significant

To evaluate whether the effect of phospho-JMJD1A on the induction of beige-selective genes in scWAT depot is cell autonomous, we isolated primary stromal vascular function (SVFs) from scWAT, and beige adipocyte differentiation was induced using ROS (hereafter called scWAT culture), as previously reported^7,24^. NE-induced S265 phosphorylation of JMJD1A in scWAT culture from *WT* mice (WT scWAT culture), but not *Jmjd1a*-S265A^KI/KI^ mice (S265A knock-in scWAT culture), as shown by immunoblot analysis with a pS265-JMJD1A-specific antibody (Fig. 3a). Both cultures differentiated with equivalent efficiency into mature lipid droplet-containing adipocytes (Fig. 3b, inset); however, as in scWAT of *Jmjd1a*-S265A^KI/KI^ mice, S265A knock-in scWAT cultures had an impaired response to NE because the expression of *Ucp1* (both mRNA and protein) along with RNAs encoding other beige-selective genes (e.g. *Cidea*, *Pgc1a*, *Prdm16*, *Irf4*, *Ppara*, *Cox8b*, and *Cpt1b*) were all reduced (Fig. 3b, c). Consistent with the reduced thermogenic response, levels for *Pgc1a* (mRNA and protein), mitochondrial-specific transcripts (*Nd1*, *2* and *4*, *Cytb*, *Cox1-3*, *Atp6*, and *8*) (Supplementary Fig. 3a), and proteins of mitochondrial oxidative phosphorylation (Fig. 3c) were all markedly reduced in the scWAT from *Jmjd1a*-S265A^KI/KI^ mice. Staining with MitoTracker Green, a marker of mitochondrial content (Fig. 3d), and mitochondrial DNA content by quantitative real-time PCR (qPCR), were also significantly lower in *Jmjd1a*-S265A^KI/KI^ scWAT, relative to *WT* controls (Fig. 3e). Additionally, in electron micrographs, scWAT from *WT* mice were packed with mitochondria containing well-ordered cristae, whereas S265A knock-in adipocytes had a paucity of mitochondria (Fig. 3f). *Jmjd1a*-S265A^KI/KI^ scWAT also had reduced basal, maximal, and uncoupling OCRs, lower NE-stimulated OCR measured by flux analyzer (Fig. 3g and Supplementary Fig. 3b), and lower NE-stimulated glycerol release (Fig. 3h). Taken together, these data show that pS265-JMJD1A contributes to beige adipocyte formation in a cell autonomous manner.

### Phosphomimetic S265D-JMJD1A induces beige-selective genes

To further investigate the molecular mechanisms of pS265-JMJD1A on the induction of beige-selective genes, we introduced WT or mutant versions of human JMJD1A (hJMJD1A) into immortalized (im) mouse preadipocytes^7,24^ (Supplementary Fig. 1d–g), where endogenous JMJD1A was pre-knocked down by short-hairpin RNA (shRNA) (Supplementary Fig. 3c). shRNA efficiently depleted endogenous JMJD1A without affecting the lipid accumulation (Supplementary Fig. 3d). Retroviruses expressing either the WT-hJMJD1A or the S265A proteins were introduced, and we showed that the levels of ectopic hJMJD1A expression were comparable to native mouse JMJD1A (Supplementary Fig. 3e). The S265A virus did not affect the lipid accumulation, and although it was similar to the studies from scWAT from the *Jmjd1a*-S265A^KI/KI^ mice above, the mutation did impair the OCR (NE-stimulated, maximal, and uncoupling), NE-stimulated glycerol release, mitochondrial content, and resulted in reduced expression of *Ucp1* mRNA and protein, along with lower levels of encoding, and other beige-selective and mitochondrial-specific mRNAs (Supplementary Fig. 3f–m).

We also made a retrovirus expressing an S265-phosphomimetic JMJD1A (S265D-hJMJD1A), in which the negatively charged aspartyl residue mimics a negatively charged phosphate group. In contrast to the S265A mutation, when the S265D virus was introduced into the JMJD1A-depleted adipocytes, it resulted in dramatically elevated constitutive expression of a distinct set of beige-selective genes (Fig. 3i). UCP1 and PGC1α protein levels were also increased (Fig. 3j). By contrast, the expression of general adipogenic genes did not differ significantly between WT and S265D-transduced cultures (Fig. 3i). Consistent with the increased expression of *Pgc1a*, *Irf4*, and *Tfam*, which are involved in mitochondrial biogenesis and function^25^, S265D transduction resulted in an increase in the constitutive levels of mitochondrial DNA (Fig. 3k), mitochondrial-specific transcripts (Supplementary Fig. 3n), and the relative amounts of proteins of the mitochondrial oxidative phosphorylation complexes (Fig. 3j). Taken together, these loss-of-function and gain-of-function data demonstrate that pS265-JMJD1A is essential for inducing the differentiation of beige adipocytes (mediated by TZDs) under chronic β-adrenergic signaling.

### TZD-induced beige adipogenesis is mediated by p-JMJD1A

**Fig. 4:**
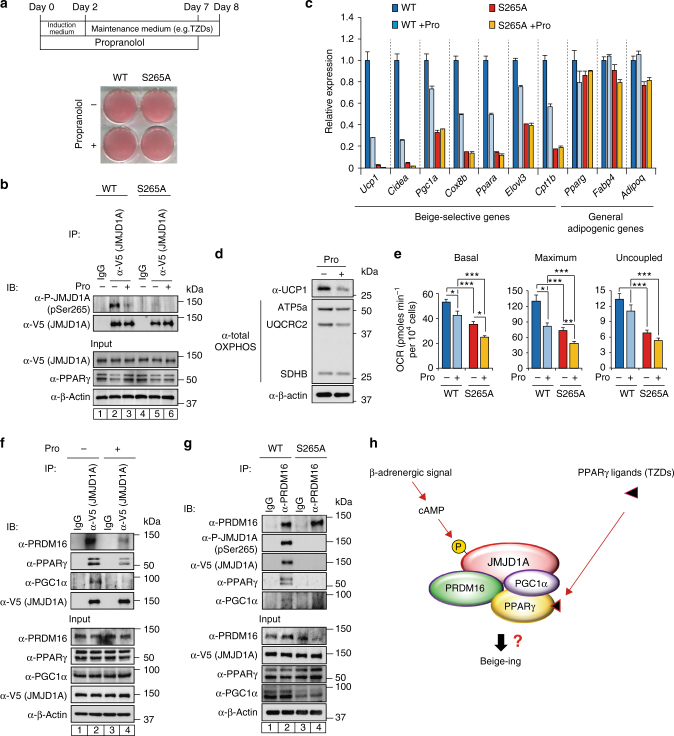
β-Adrenergic signal is required for the induction of beige-selective genes mediated by PPARγ ligand. **a** WT-hJMJD1A-transduced or S265A-hJMJD1A-transduced im-scWATs were differentiated for beige adipogenesis in the presence or absence of propranolol (Pro), as schematically illustrated (top), and ORO staining was performed (bottom). **b** Whole-cell lysates (WCL) from WT-hJMJD1A-transduced or S265A-hJMJD1A-transduced im-scWATs differentiated under Pro (100 nM) plus or minus condition were subjected to immunoprecipitation (IP) with anti-V5 antibody, followed by immunoblot (IB) analysis with anti-P-JMJD1A (pSer265) antibody. **c** qPCR analysis of beige-selective genes and general adipogenic genes in WT-hJMJD1A-transduced or S265A-hJMJD1A-transduced im-scWATs under Pro plus or minus condition (mean ± s.e.m. of three technical replicates). **d** Immunoblotting with anti-UCP1 or anti-total OXPHOS antibodies cocktail using WCL from indicated viral transduced im-scWATs, differentiated under Pro plus or minus condition. **e** OCRs (basal, maximum, and uncoupled) of WT-hJMJD1A-transduced or S265A-hJMJD1A-transduced im-scWATs, differentiated under Pro plus or minus condition. Data are mean ± s.e.m. of five technical replicates. **f**, **g** Immunoblotting with anti-PRDM16, anti-P-JMJD1A, anti-V5, anti-PPARγ, or anti-PGC1α antibody following immunoprecipitation with anti-PRDM16 antibody (**f**) or with anti-V5 antibody for JMJD1A (**g**), from WCL of differentiated WT-hJMJD1A-transduced or S265A-hJMJD1A-transduced im-scWATs. Uncropped images of the blots (**b**, **d**, **f**, **g**) are shown in Supplementary Fig. 8. Analysis of variance was performed, followed by Tukey’s post hoc comparison in **e**. *P < 0.05, **P < 0.01, and ***P < 0.005 were considered statistically significant. **h** Schematic drawing of p265-JMJD1A-PPARγ-PGC1α-PRDM16 protein complex. Integration of β-adrenergic-cAMP signaling and the PPARγ ligand binding is mediated by JMJD1A through a mechanism where pS265-JMJD1A forms a complex with PGC1α, PRDM16, and PPARγ to mediate expressions of beige-selective genes

Chronic activation of PPARγ by TZDs such as ROS and BAR activation, both stimulate beigeing in mammals (reviewed in refs. ^3,21^). However, whether or not BAR activation is required for beige fat cell development by TZDs is not clearly understood. We previously showed that JMJD1A integrates β-adrenergic-cAMP signaling with the PPARγ gene activation program through a mechanism where pS265-JMJD1A performs a scaffolding role to activate key PPARγ target genes required for thermogenesis in BAT^13^. Because the fetal bovine serum (FBS) used for cell culture contains non-negligible levels of catecholamines^13^, we included the BAR blocker propranolol (Pro) during differentiation. Regardless of Pro treatment, preadipocytes differentiated into adipocytes with similar lipid accumulation (Fig. 4a). Pro reduced the β-adrenergic signaling because Pro blunted the phosphorylation of JMJD1A on S265 (Fig. 4b, compare lanes 2 and 3), and Pro also markedly reduced the expression of beige-selective genes (Fig. 4c), protein levels of UCP1, and mitochondrial oxidative phosphorylation complex subunits (Fig. 4d), along with reduced OCR (Fig. 4e). Furthermore, the expression of beige-selective genes was markedly reduced in S265A-hJMJD1A-transduced cultures, while similar levels of general adipogenic genes were expressed in both WT-transduced and S265A-hJMJD1A-transduced cultures, regardless of the Pro treatment (Fig. 4c). These data show that chronic β-adrenergic stimulation is essential for TZD-induced beige-selective activation via the downstream pS265-JMJD1A.

### pS265-JMJD1A-PPARγ-PGC1α-PRDM16 protein complex

PRDM16 plays a central role in BAT/beige adipogenesis through interacting with protein complexes containing transcription (co-) factors (e.g., PPARγ, C/EBPβ, PGC1α) and an epigenetic thermogene enzyme EHMT1 (reviewed in refs. ^2,3,21^). EHMT1 epigenetically silences the gene expression through monomethylation and dimethylation of H3K9 of chromatin associated with target genes during brown adipogenesis; however, how PRDM16 activates silenced genes involved in thermogenesis in scWAT is not well understood. The genome-wide DNA-binding analysis of JMJD1A and PRDM16 in brown adipocytes using ChIP-seq data reported by us and others^13,26^ showed that approximately 48% of PRDM16 binding sites overlapped with JMJD1A binding sites following β-adrenergic stimulation (Supplementary Fig. 4a), and the sites of overlap include several beige-selective genes (Supplementary Fig. 4b). Because PRDM16 associates with several proteins, including PPARγ and PGC1α (reviewed in ref. ^3^), and pS265-JMJD1A forms a protein complex with PPARγ^13^, we hypothesized that pS265-JMJD1A may interact with PRDM16 together with PPARγ and PGC1α.

A retrovirus-expressing JMJD1A tagged with a V5 peptide was transduced into the im-JMJD1A knockdown adipocytes used above. We then immunoprecipitated the tagged JMJD1A in WT-hJMJD1A-transduced adipocytes after beige conversion, and examined JMJD1A and putative proteins through co-immunoprecipitation with antibodies against PRDM16, PPARγ, and PGC1α. The result showed that JMJD1A co-immunoprecipitated with PRDM16 together with PPARγ and PGC1α in the absence of Pro (Fig. 4f, compare lanes 1 and 2), and the levels of the signature beige proteins (i.e., PRDM16, PGC1α, and PPARγ) in the co-immunoprecipitates were greatly reduced when Pro was included in the culture medium (Fig. 4f, compare lanes 2 and 4). Consistent with previous reports^27,28^, PPARγ and PGC1α were co-immunoprecipitated with native PRDM16 in WT-hJMJD1A-transduced adipocytes, but not in S265A-hJMJD1A-transduced adipocytes (Fig. 4g). There was also little evidence of interaction between the phosphorylation-defective S265A-JMJD1A and PPARγ, PGC1α, or PRDM16 (Supplementary Fig. 4c). In a reciprocal manner, much stronger signals for signature beige proteins were detected by immunoblot analysis of the immunoprecipitates of V5-tagged phosphomimetic S265D-hJMJD1A-transduced adipocytes, compared to those of WT-hJMJD1A adipocytes (Supplementary Fig. 4d). The association of pS265-JMJD1A and signature beige proteins were also confirmed in primary adipocytes, differentiated from SVF of *WT* and S265A^KI/KI^ scWATs (Supplementary Fig. 4e). Collectively, these results demonstrate that persistent β-adrenergic signaling mediates the formation of a multi-protein complex containing JMJD1A-PPARγ-PGC1α-PRDM16, and that phosphorylation of S265 in JMJD1A nucleates formation of the entire complex, which is essential for beige-selective gene induction in scWAT (Fig. 4h).

A common DNA-binding motif in ChIP-seq peaks for all the three, JMJD1A, PRDM16, and PPARγ, following BAR activation, and includes the binding motif for EBF, a key factor for browning adipocyte identity^13,29^ (Supplementary Fig. 4f). These data also suggest that beige-selective gene inducing effects by PPARγ and PRDM16 are, at least in part, mediated by JMJD1A. Additionally, because H3K9me2 levels are constitutively high in scWAT and decline during beige conversion, we hypothesized that pS265-JMJD1A-PPARγ complex might contribute to the beige cell function through erasing the nucleosomal H3K9me2 of beige-selective genes to convert them to a euchromatin-like state.

### pS265-JMJD1A demethylates H3K9me2 on beige-selective genes

**Fig. 5:**
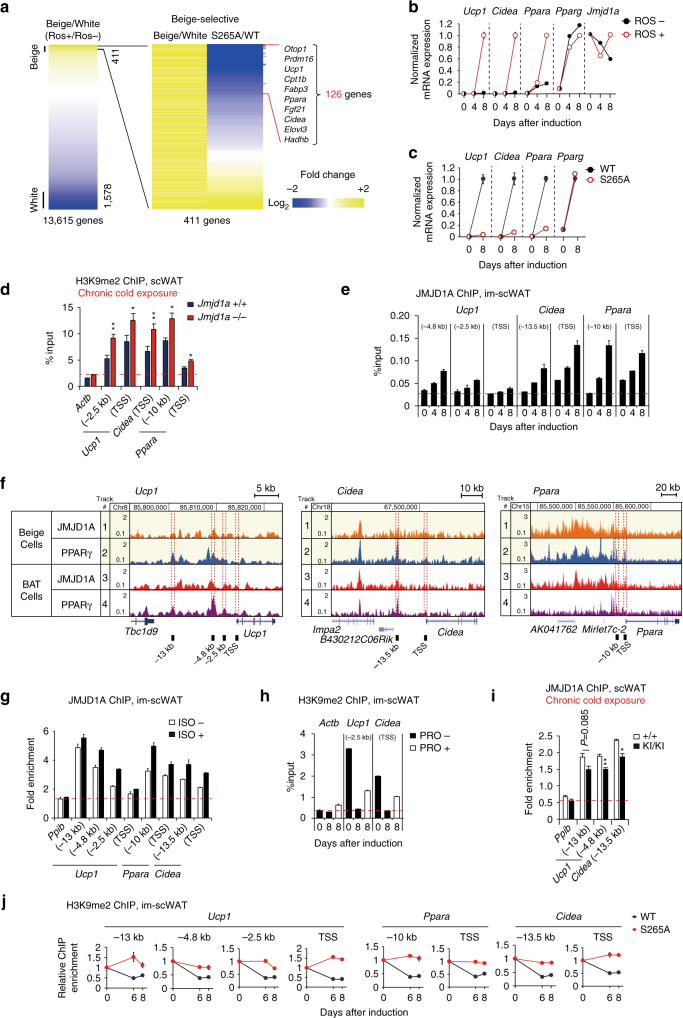
JMJD1A demethylates H3K9me2 on beige-selective genes. **a** RNA-seq heat map depicting expression ratio comparison between beige and white adipocytes, differentiated from im-scWATs under rosiglitazone (ROS) plus or minus condition (left). RNA-seq heat map of 411 beige-selective genes from the left panel depicts comparison of expression ratio between WT-hJMJD1A-transduced and S265A-hJMJD1A-transduced im-scWATs (right). Changes are log_2_ expression ratios of FPKM, as indicated in a color intensity scale. **b**, **c** qPCR analysis of beige-selective genes and *Pparg* in im-scWATs differentiated with or without ROS (**b**) or differentiated WT-hJMJD1A-transduced or S265A-hJMJD1A-transduced im-scWATs (**c**). **d** H3K9me2 ChIP-qPCR on beige-selective genes using scWAT from age-matched *WT* (+/+) and *Jmjd1a*-null (−/−) mice placed at 4 °C for 1 week (*n* = 4 per genotype group). **e** JMJD1A ChIP-qPCR on beige-selective genes during ROS-induced beige adipogenesis in im-scWATs. **f** ChIP-seq profiles for JMJD1A and PPARγ on *Ucp1*, *Cidea*, and *Ppara* genomic regions in differentiated im-scWATs (beige cells) and im-BATs (BAT cells). **g**, **h** ChIP-qPCR showing isoproterenol (ISO) treatment increased JMJD1A recruitment in differentiated im-scWATs (**g**) and the decrease of H3K9me2 levels in beige-selective genes in differentiated beige adipocytes by ROS was blunted by Pro treatment (**h**). **i** JMJD1A ChIP-qPCR on beige-selective genes in scWAT of *WT* and *Jmjd1a*-S265A^KI/KI^ mice following 1-week cold exposure (*WT*: *n* = 3; *Jmjd1a*-S265A^KI/KI^: *n* = 6). **j** ChIP-qPCR showing the decrease of H3K9me2 levels on indicated beige-selective genes during beige adipogenesis is impaired in S265A-hJMJD1A-transduced im-scWATs. The signal in day 0 of differentiation is set as 1. Data are mean ± s.e.m. of three technical replicates in a representative experiments performed at least three times (**b**, **c**, **e**, **g**, **h**, **j**). Student’s *t* test was performed for comparisons in **d**,** i**. *P < 0.05 and **P < 0.01 were considered statistically significant

To evaluate the role of JMJD1A during beige adipogenesis on a genome-wide level, we analyzed the global transcriptional changes associated with beige adipogenesis by performing RNA-sequencing (RNA-seq) in im-scWAT preadipocytes and im-scWAT adipocytes (Supplementary Fig. 1d–g)^7,24^ treated with or without ROS to induce white or beige fat cells, respectively (Fig. 5a). There were 13,615 expressed genes in white and beige adipocytes, and a total of 1989 were “differentially” expressed (here, a “differentially” expressed gene is defined as a transcript with a fragment per kilobase of exon per million fragments mapped (FPKM) in beige adipocytes altered by two-fold, relative to that in white adipocytes), of these 411 are beige-selective and 1578 are white-selective. Notably, several known white-selective and beige-selective genes^30^ were differentially expressed between white and beige im-scWAT adipocytes (Supplementary Fig. 5a). qPCR confirmed that beige-selective genes (*Ucp1*, *Cidea*, and *Ppara*) were robustly induced during beige adipogenesis (Fig. 5b). Comparison of WT-transduced and S265A-hJMJD1A-transduced adipocytes revealed a total of 126 genes out of 411 beige-selective genes that were not induced by the S265A mutation in JMJD1A (Fig. 5a). These include *Otop1*, *Prdm16*, *Ucp1*, *Cpt1b*, *Ppara*, *Fgf21*, *Cidea*, *Elovl3*, and *Hadh1b*, but not the general adipogenic gene, *Pparg* (Fig. 5a, c). A gene ontology (GO)-enrichment analysis revealed that these beige-selective genes were highly associated with GO terms related to mitochondrial component (Supplementary Fig. 5b, left) and functions including lipid metabolic processes and fatty acid oxidation (Supplementary Fig. 5b, right).

**Fig. 6:**
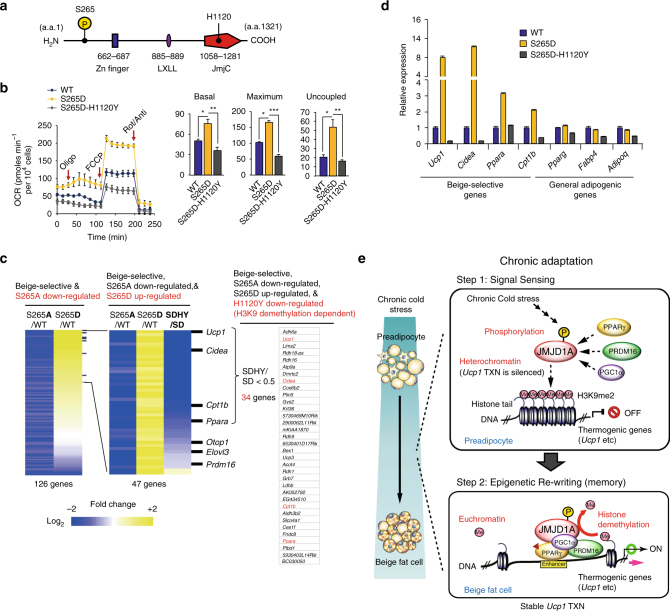
Demethylation activity is pivotal for beige-selective gene inductions. **a** Schematic representation of the domain structure of human JMJD1A. Phosphorylation site at S265 and Fe(II) binding site at H1120 are shown. **b** OCR in im-scWATs stably expressing WT-hJMJD1A, S265D-hJMJD1A, or S265D-H1120Y-hJMJD1A treated sequentially with oligomycin (Oligo), FCCP, and rotenone/antimycin A (Rot/Anti) (left). Basal, maximum, and uncoupled respiration calculated from the left (right). Data are mean ± s.e.m. of three technical replicates in a representative experiment. Analysis of variance were performed, followed by Tukey’s post hoc comparison. *P < 0.05, **P < 0.01, and ***P < 0.005 were considered statistically significant. **c** Heat map of mRNA levels determined by RNA-seq analysis of the indicated WT or mutant versions of hJMJD1A expressing im-scWATs. Changes are log_2_ expression (FPKM) ratios. For reference, a color intensity scale is included. Thirty-four genes that are beige-selective, S265A downregulated, S265D upregulated, and H1120Y downregulated (H3K9 demethylation-dependent) are listed (right). **d** qPCR analysis confirming RNA-seq shown in **c**. Mean ± s.e.m. of three technical replicates. **e** Hypothetical model. Cold stimulated β-adrenergic signal leads to phosphorylation of JMJD1A (Step 1), which triggers the formation of a PRDM16-PGC1α-PPARγ transcription complex that targets beige-selective genes (Step 1, top). JMJD1A then demethylates H3K9me2 to turn on the transcription of these genes in scWAT (Step 2, bottom)

Given that β-adrenergic signaling results in phosphorylation of S265 in JMJD1A triggering the formation of a complex containing JMJD1A-PPARγ-PGC1α-PRDM16 (step 1 in our model: signal sensing) (Fig. 4h), we hypothesized that JMJD1A may subsequently demethylate the neighboring repressive histones at H3K9me2 on the thermogenic gene enhancer/promoter regions (step 2 in our model: epigenetic re-writing). We further proposed that this step 2 may establish a stable gene expression program for beigeing that would protect the mammals from chronic cold exposure (see Fig. 6e, shown below). H3K9me2 levels are high before cold exposure and are decreased during chronic cold exposure in scWAT (Fig. 1f, g, i). The H3K9me2 levels on the *Ucp1*, *Cidea*, and *Ppara* gene enhancer/promoter regions were significantly higher in scWAT of cold-acclimated *Jmjd1a-*null mice (Fig. 5d), suggesting that JMJD1A-mediated H3K9me2 demethylation is pivotal for the activation of beige-selective gene expression during beigeing of inguinal scWAT. In cultured scWAT beige adipogenesis, H3K9me2 levels were decreased toward the end of differentiation, as shown (Fig. 1g). Correlatively, expression of the associated genes (e.g., *Ucp1*, *Cidea*, and *Ppara*) was increased (Fig. 5a, b), and the JMJD1A recruitment on these genes was increased toward the end of differentiation, as assessed by ChIP-qPCR (Fig. 5e). ChIP-seq of JMJD1A and PPARγ in im-scWATs, subjected to the beige differentiation protocol, revealed that JMJD1A is recruited to the enhancer/promoter regions of beige-selective genes (e.g., *Ucp1*, *Cidea*, and *Ppara*) co-localizing with PPARγ during conversion to beige cells (Fig. 5f). In agreement with β-adrenergic signal-induced multi-protein complex formation (Fig. 4f, g, h and Supplementary Fig. 4c–e), JMJD1A recruitment was increased by the treatment with BAR agonist isoproterenol (ISO) (Fig. 5g) and, reciprocally, β-blocker Pro treatment prevented the reduction of H3K9me2 levels on these target genes (Fig. 5h), in agreement with the prevention of these beige-selective gene inductions (Fig. 4c). The key role of S265 phosphorylation of JMJD1A was further demonstrated in vivo: the recruitment of JMJD1A to enhancers and promoters of *Ucp1* and *Cidea* was significantly reduced in scWAT of *Jmjd1a*-S265A^KI/KI^ mice, compared to *WT* mice following chronic cold exposure (Fig. 5i).

To determine whether phosphorylation of S265 on JMJD1A is required for H3K9me2 demethylation, we examined the levels of H3K9me2 during beige adipogenesis of WT-hJMJD1A-transduced vs. S265A-hJMJD1A-transduced preadipocytes. H3K9me2 levels on *Ucp1*, *Cidea*, and Ppara gene enhancer/promoter regions were reduced at days 6 and 8 of differentiation in WT-hJMJD1A-transduced im-scWAT preadipocytes, while H3K9me2 levels were maintained at high levels in the phosphorylation-defective S265A-hJMJD1A-transduced cells, as evidenced by ChIP (Fig. 5j). The changes in H3K9me2 were consistent with the gene expression profiles (Fig. 5c). In addition, in vitro H3K9 demethylation enzymatic activities between WT-hJMJD1A and S265A-hJMJD1A proteins do not differ^13^. These data provide strong support for the two-step model, where β-adrenergic signaling-dependent phosphorylation of S265 occurs first and is required for JMJD1A-mediated H3K9me2 demethylation on the thermogenic genes.

### Demethylation by JMJD1A is pivotal for beige adipogenesis

The decrease in H3K9me2, that occurs upon recruitment of JMJD1A in scWAT, suggests that the catalytic activity of JMJD1A might be directly required for demethylation associated with the induction of beige-selective gene expression in scWAT. To directly analyze this, we transduced WT, S265D, or the double S265D-H1120Y mutant of JMJD1A into im-scWAT, where endogenous JMJD1A was knocked down (Fig. 6a and Supplementary Fig. 6a). The H1120Y substitution changes the catalytic histidine from the carboxyl terminal demethylase domain into tyrosine, rendering the protein catalytically dead^13^. As shown earlier, the phosphomimetic S265D-hJMJD1A functions as a constitutive gain-of-function mutant that induces beige-selective genes by facilitating the protein complex with PGC1α-PPARγ-PRDM16 (Fig. 3i–k and Supplementary Fig. 4d). Therefore, S265D-H1120Y double mutant is an ideal tool to examine the necessity of catalytic activity of JMJD1A for the induction of beige-selective genes. As shown in Fig. 6b, transduction of S265D-hJMJD1A robustly induced basal, maximal, and uncoupled respiration; by contrast, transduction with the double mutant S265D-H1120Y severely abrogated the S265D-induced respiration in beige adipocytes. These data indicate that demethylation activity of JMJD1A is absolutely required for promoting thermogenic function in the induction of beige-selective genes.

To comprehensively elucidate beige-selective genes that are dependent on both JMJD1A S265 phosphorylation and demethylation activity, we compared RNA-seq transcriptome data of WT-, S265D-, and S265D-H1120Y-hJMJD1A -transduced adipocytes. As shown in Fig. 6c, of 126 genes that are both beige-selective and reduced in the S265A mutant cells (Fig. 5a), 47 of these genes were upregulated in S265D-hJMJD1A-transduced cultures (Fig. 6c). The expression of 34 genes of these 47 genes, including *Ucp1*, *Cidea*, *Cpt1b*, *Ppara*, and so on, were reduced by at least half when the catalytically dead double mutant S265D-H1120Y-hJMJD1A was transduced, relative to the S265D-constitutively active JMJD1A (FPKM score; SDHY/SD < 0.5) (Fig. 6c and Supplementary Fig. 6b). These 34 genes are considered to mediate cellular memory because their expressions are highly dependent on demethylation activity of JMJD1A. The expression of additional 13 genes including *Otop1*, *Elov3*, and *Prdm16* were also reduced, but not as much as *Ucp1* and *Cidea* (Fig. 6c and Supplementary Fig. 6c). The expression differences for some of the key genes was confirmed by qPCR (Fig. 6d). Collectively, these results demonstrate that persistent cold exposure or β-adrenergic agonist stimulation induces beige adipogenesis via two steps: β-adrenergic signal sensing, followed by H3K9me2 demethylation (Fig. 6e).

### Insulin resistance phenotype of *Jmjd1a*-S265A^KI/KI^ mice

**Fig. 7:**
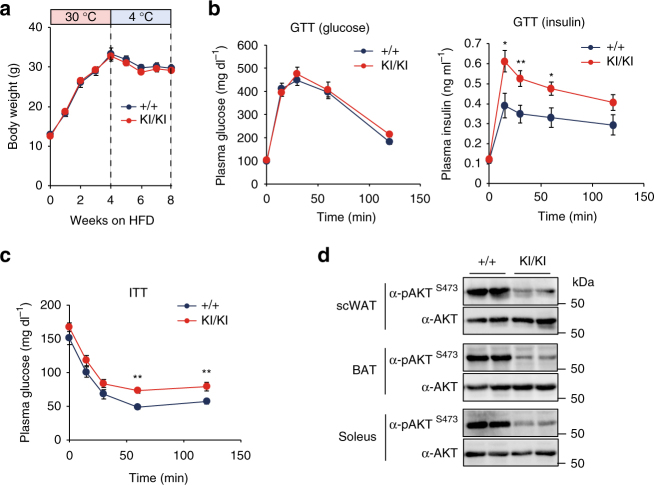
Insulin resistance phenotype of *Jmjd1a*-S265A^KI/KI^ mice. **a** Body weight changes. *WT* (+/+) and *Jmjd1a*-S265A^KI/KI^ mice (*WT*: *n* = 6; *Jmjd1a*-S265A^KI/KI^: *n* = 8) were fed on HFD for 4 weeks at 30 °C, and then switched to 4 °C for 4 weeks. **b**,** c** Glucose tolerance test (GTT) (**b**) and insulin tolerance test (ITT) (**c**) in each genotype group mice fed on HFD after cold acclimation in **a** (*WT*: *n* = 6; *Jmjd1a*-S265A^KI/KI^: *n* = 7). **d** Assessment of insulin signaling as quantified by the phosphorylation of AKT-S473 in scWAT, BAT, or soleus from each genotype mice fed on HFD after cold acclimation presented in **a**. Uncropped images of the blots are shown in Supplementary Fig. 8. Data are mean ± s.e.m. (**a**–**c**) Student’s *t* test was performed for comparisons in **a**–**c**. *P < 0.05 and **P < 0.01 were considered statistically significant

Beige adipocyte activity affects the systemic metabolism and significantly contributes to the whole-body insulin sensitivity^6,31,32^. Therefore, we investigated whether abrogation of beige fat recruitment in *Jmjd1a-*S265A^KI/KI^ mice also results in global metabolic dysfunction. For these studies, *WT* and *Jmjd1a-*S265A^KI/KI^ mice were fed on a high-fat diet (HFD) and housed at 30 °C for 4 weeks, and then switched to 4 °C for an additional 4 weeks on the HFD. Body weight gain and glucose tolerance were similar in *WT* and *Jmjd1a*-S265A^KI/KI^ mice (Fig. 7a). However, insulin levels in the *Jmjd1a*-S265A^KI/KI^ mice were higher than *WT* mice during the glucose tolerance test (GTT), despite their comparable glucose levels (Fig. 7b). Consistently, insulin tolerance test (ITT) demonstrated that insulin-mediated suppression of plasma glucose was significantly impaired in *Jmjd1a*-S265A^KI/KI^ mice (Fig. 7c). Glucose and insulin levels during the GTT did not differ between *WT* and *Jmjd1a-*S265A^KI/KI^ mice before cold acclimation (Supplementary Fig. 7a), indicating that HFD-fed *Jmjd1a-*S265A^KI/KI^ mice have impaired insulin signaling only when the animals encounter the additional stress of cold exposure. This was further confirmed by insulin signaling studies, which revealed the loss of S265 phosphorylation impaired insulin action, as quantified by the phosphorylation of S473 on AKT in scWAT, BAT, and soleus muscle (Fig. 7d). These systemic differences are likely to be due, at least in part, to the reduced beige recruitment and activity in the HFD-fed cold-acclimated *Jmjd1a*-S265A^KI/KI^ mice, because there was no significant difference in insulin action at RT (Supplementary Fig. 7b).

## Discussion

**Fig. 8:**
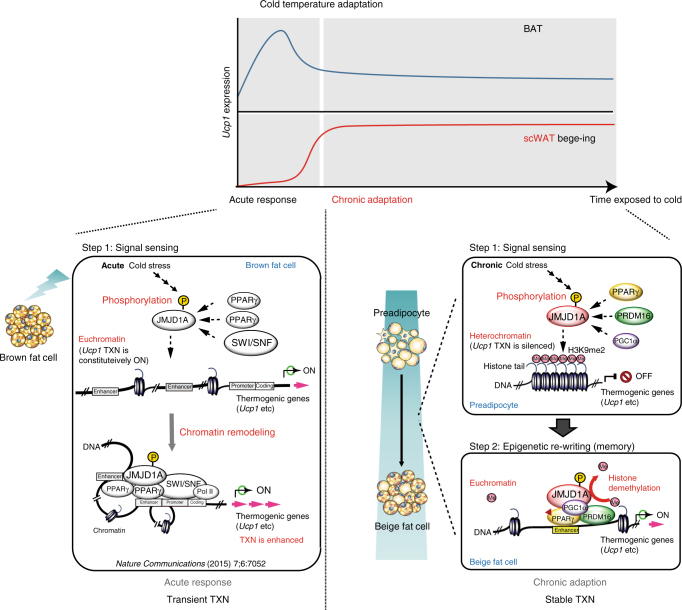
Complementary mechanisms for thermogenic gene induction in acute and chronic cold stress via overlapping, but distinct mechanisms of JMJD1A. Brown fat cells mediate acute and robust thermogenic activation of *Ucp1*, while scWAT-derived beige fat cells contribute to an adaptive response against chronic cold exposure (top). The acute response in BAT requires a BAR-dependent phosphorylation of JMJD1A that facilitates long-range enhancer-promoter interactions and stimulate thermogenic gene expressions, but this does not require the intrinsic H3K9me2 demethylation activity of JMJD1A (left bottom). The chronic adaptation in beigeing requires both phosphorylation-dependent chromatin recruitment and H3K9me2 demethylation activity of JMJD1A (right bottom). These histone demethylation-independent acute *Ucp1* induction in BAT and demethylation-dependent chronic *Ucp1* expression in beige scWAT ensure an ordered transition between acute and chronic adaptation to cold stress. TXN transcription

Beige and brown adipocytes have thermogenic activity, but they are postulated to have complementary functions in the maintenance of energy balance and thermogenesis^2^. BAT mediates acute and robust thermogenic activity, while scWAT-derived beige adipocytes contribute to an adaptive response against chronic cold exposure. It is likely that the combined action of *Ucp1*-dependent thermogenesis in BAT and beige scWAT is physiologically important to ensure acute and chronic BAR-dependent uncoupled respiration for protection from the cold environmental challenges, where BAR-dependent uncoupling is needed (Fig. 8, top). We show that these complementary mechanisms for thermogenic gene induction in acute and chronic cold stress occur via overlapping, but distinct mechanisms (Fig. 8, bottom). The acute response in BAT requires a BAR-dependent phospho-switch in JMJD1A, resulting in its recruitment to thermogenic gene chromatin that is independent of changes in H3K9 methylation. In contrast, the chronic adaptation in beige adipocytes requires both the phospho-switch-dependent chromatin recruitment and H3K9 demethylation activities of JMJD1A.

The present results also provide a prototypic mechanism for how an epigenetic modifier senses chronic environmental stress via signal-specific post-translational modification (PTM) (step 1), and then re-writes the epigenetic information (step 2) to induce an adaptive phenotype in response to cold stress. Signal-dependent PTMs are also found in many transcription factors and co-factors that reversibly associate with chromatin through noncovalent interactions to mediate dynamic genome events (reviewed in refs. ^3,33^). Additionally, the epigenetic modifiers can also provide stable-covalent modifications of chromatin to ensure that the gene expression changes in response to stress signaling are sustained over time. The mechanism described here is in response to cold stress, but it is likely that similar mechanisms are involved in adaptive changes to additional chronic environmental stresses, including nutritional variation, fasting/caloric restriction, pathogen exposure, and chemical stress.

Beige fat cells also actively consume fat and glucose to contribute to the whole-body insulin sensitivity and help regulate the whole-body energy balance. When we exposed the *Jmjd1a*-S265A^KI/KI^ mice to HFD feeding followed by cold exposure, we also uncovered a unique role for JMJD1A-mediated beigeing in regulating the insulin sensitivity. The phosphorylation-defective knock-in mice and *WT* mice gained similar amounts of weight on the HFD and had similar responses to the GTT challenge. However, after exposure to 4 °C, mice with a S265A mutation of JMJD1A that prevents the BAR phospho-switch displayed both a hyperinsulinemic response during GTT, and had deficient glucose clearance when challenged with insulin. These responses reveal a crucial role for pS265-JMJD1A in glucose regulation only when mice are exposed to the additional stress of cold exposure on top of the HFD challenge. However, because the mice have a global knock-in for S265A, and they also show defective insulin responses in BAT and muscle, we cannot conclude that the defective insulin response is only due to the defect in scWAT beige conversion.

Induction of beige fat cells is now drawing great clinical interest in the search for therapeutic targets for metabolic disorders. The present results show that preventing phosphorylation of S265 JMJD1A results in defective thermogenesis and the development of cold-dependent insulin resistance in mice, while the expression of phosphomimetic S265D-JMJD1A promotes a beige adipogenic program. Thus, S265 phosphorylation of JMJD1A might prove to be a novel molecular target for the treatment of metabolic disorders via promoting beige adipogenesis.

## Methods

### Generation of *Jmjd1a*-S265A^KI/KI^ mice

A targeting plasmid was constructed by using genomic DNA fragments derived from RENKA mice. A neomycin cassette flanked by two loxP sites in intron 8 as 3′ homologous arm and S265A point mutation site (TCT (Ser) → GCA (Ala)) in exons 5–8 as 5′ homologous arm was introduced into the *Jmjd1a* locus of ES cells (derived from the RENKA strain). Electroporation, selection, and screening were performed with standard gene-targeting techniques. Chimeric males were generated by using the morula aggregation technique and mated to CAG-Cre female mice to obtain *Jmjd1a*-S265A^+/KI^ mice, removed the neomycin-resistant gene. After achieving germline transmission, the *Jmjd1a*-S265A^+/KI^ mice were back-crossed with C57BL/6N mice, and the C57BL/6N background *Jmjd1a*-S265A^+/KI^ mice were mated to obtain the *Jmjd1a*-S265A^KI/KI^ mice. Genotyping of mice used in this study was performed by PCR of the tail DNA followed by direct sequencing or *Pst*I digestion, as shown in Supplementary Fig. 1i, j; S265A mutation was decided when a 700 bp PCR product produced by a pair of oligonucleotides (5′-gcttggacccatctactcagtg-3′ and 5′-caagatctggaacttgtaaca-3′) was digested by *Pst*I and divided into two DNA fragments (200 and 500 bp).

### Animal experiments

All animal studies were approved by the Animal Care and Use Committee of The University of Tokyo. Mice were maintained under a 12 h light/12 h dark cycle (08:00–20:00) at constant temperature (23 °C) with free access to food and water. Animals were fed a normal chow diet (CE-2, CLEA Japan Inc.) or HFD consisting of 58.0% fat, 15.0% protein, and 27.0% carbohydrate from calories from the age of 4 weeks, as indicated^34,35^. *WT* (+/+) and *Jmjd1a*-null (−/−) mice (C57BL/6J background) were described previously^15^ and *Jmjd1a*-S265A^KI/KI^ mice are described above. For chronic cold exposure experiments, animals were single-caged and exposed to 4 °C for 1 week, unless otherwise stated. At the conclusion of experiments, tissues were harvested and snap frozen in liquid nitrogen for RNA and protein analysis or fixed in 10% formalin for histology.

To analyze the BAT thermogenic function in vivo, we examined the whole animal oxygen consumption following intraperitoneal injection of NE (1 mg kg^−1^ body weight (BW)) for 1 h, the standard activator of BAT-mediated thermogenesis. On the day of the experiment, mice were first placed in metabolic chambers at 30 °C, sedated with 2,2,2-tribromoethanol (36 mg kg^−1^ BW, T48402; Sigma), and followed 15 min later by NE injection. Energy expenditure was measured using indirect calorimetry (MK-5000RQ; Muromachi)^13^. The chamber volume was 720 ml, and the airflow to the chamber was 400 ml min^−1^ for 30 °C-acclimated mice and 800 ml min^−1^ for 4 °C-acclimated mice. Samples were taken every 3 min, and standard gas reference was taken every 30 min.

Details of the age and sex of mice used are shown in Supplementary Table 1.

### Body temperature

Core body temperature was monitored using a rectal thermometer (Bio Research Center; BDT-100C). Mice were acclimated to thermoneutrality (30 °C) for 4 weeks, prior to the experiments of acute cold exposure (Fig. 1j). The body temperature was measured at 8–9 weeks of age in control and *Jmjd1a*-S265A^KI/KI^ mice at 10 a.m.

### Plasma parameters

Plasma glucose concentrations were measured by Glucose C2 test (Wako). The plasma insulin level was determined by enzyme-linked immunosorbent assay using an Insulin Immunoassay Kit (Shibayagi), according to the manufacturer’s instructions.

### Glucose and ITTs

For GTT, mice were fasted overnight and then given an intraperitoneal injection of glucose (2 g kg^−1^ BW)^15^. For ITT, non-fasted mice were injected intraperitoneally 4 h after the beginning of the light cycle with 0.4 U kg^−1^ BW of human insulin (Humalin^®^ R, Eli Lilly & Co., Japan). Blood samples for glucose and insulin measurements were obtained from the tail vein at indicated times. For AKT-S473 phosphorylation, all tissues were harvested 10 min after inferior vena cava injection of insulin (0.03 unit per head) under anesthesia with 2,2,2-tribromoethanol (36 mg per kg BW).

### Antibodies

Mouse monoclonal antibodies immunoglobulin G-F1411 (IgG-F1411) and IgG-F1430 against mouse PRDM16 were produced by immunizing mice with affinity-purified recombinant full-length mouse PRDM16 protein. Mouse monoclonal antibody IgG-F0618 against mouse JMJD1A (amino acids 843–893) was produced by immunizing mice with gp64 fusion protein, expressing baculovirus^13^. Mouse monoclonal antibodies IgG-F1628 against hJMJD1A were produced by immunizing mice with affinity-purified recombinant full-length hJMJD1A protein^13^. A rabbit polyclonal phospho-specific antibody against JMJD1A S265 was produced with a synthetic phosphopeptide, corresponding to residues surrounding Ser265 JMJD1A (Ac-C-KRKS(pS)ENNGS-amide)^13^. A list of other antibodies used in this article is shown in Supplementary Table 2.

### Primary adipocyte cultures from scWAT and BAT

SVF from inguinal scWAT was obtained from 3–6-week-old mice, as described^7,24^. Briefly, scWATs were first washed in phosphate-buffered saline (PBS) before being subjected to enzymatic digestion with 1.5 mg ml^−1^ collagenase D (Roche) and 2.4 U ml^−1^ dispase II (Roche) for 40 min at 37 °C to obtain a single-cell suspension. After digestion, the centrifuged cell pellet, termed SVF, was resuspended in the complete medium (Dulbecco’s modified Eagle’s medium/F12 (DMEM/F12) (Sigma), containing 1.2 g l^−1^ NaHCO_3_ and 10% FBS) before serial filtration through a 100 μm nylon cell strainer (BD Falcon). To remove the red blood cells, immune cells, and other contaminants, SVFs were plated in collagen-coated plates, and 1–2 h later, the medium was aspirated, washed with PBS twice, and then fresh medium was added. Primary brown adipocytes from SVFs were fractionated and cultured according to published methods^7,24^. SVFs from interscapular BAT were isolated from 1–3-day-old mice.

### Adipocyte differentiation

Induction for beige adipocyte from inguinal scWAT was performed as described previously^7,24^, with modification as follows. Briefly, primary preadipocytes cultured to ~100% confluence in complete medium were induced for differentiation with induction medium (i.e., complete medium containing 0.125 mM indomethacin, 5 μM dexamethasone, 0.5 mM 3-isobutyl-1-methylxanthine, 0.5 μM ROS, 5 μg ml^−1^ insulin, and 1 nM T3) (day 0). After 2 days, cells were incubated in the maintenance medium with 0.5 μM ROS (i.e., complete medium containing only insulin, T3, and ROS) for another 5 days. On day 7, unless otherwise stated, the medium was switched to maintenance medium without ROS (i.e., complete medium containing only insulin and T3) and cultured for another 1 day before the assay (day 8).

im-scWAT preadipocytes were derived from inguinal scWATs that were established by infecting isolated SVFs of inguinal WAT with retrovirus-expressing large T antigen pBabe SV40 Large T antigen from Addgene (no. 13970)^7,24^. For beige adipocyte induction, im-scWAT preadipocytes were cultured in DMEM supplemented with 10% FBS at 37 °C in 5% CO_2_ until confluent (day 0), and then treated with the culture medium containing 0.125 mM indomethacin, 5 μM dexamethasone, 0.5 mM 3-isobutyl-1-methylxanthine, 0.5 μM ROS, 5 μg ml^−1^ insulin, and 1 nM T3 for 48 h (day 2), followed by treatment with only insulin, T3, and ROS for another 6 days, with medium replacement every 2 days (day 8)^7,24^.

For brown adipocyte induction from SVF of BATs, cells were induced for differentiation with DMEM/HAMF12 medium, containing 5 μg ml^−1^ insulin, 1 nM T3, 0.125 mM indomethacin, 5 μM dexamethasone, and 0.5 mM 3-isobutyl-1-methylxanthine for 2 days. From day 2, cells were cultured only in the presence of insulin and T3.

### BAR activation and antagonist treatment

To activate BAR in tissue culture experiment, NE (10 μM for 1–2 h) or nonselective BAR agonist ISO (1 μM) was added to the culture medium. To block BAR activation, 100 nM of Pro was added, as described under the legends for figures. In the mice study, 1 mg kg^−1^ BW of NE was intraperitoneally injected, as described under Animal Experiments.

### Oil red O staining

Cells at the specified stage of differentiation were rinsed with PBS and fixed with 3.7% formaldehyde in H_2_O for 10 min. After two washes in PBS and one wash with 60% isopropanol for 1 min, cells were stained for 15 min in freshly diluted ORO solution (0.18% (wt vol^−1^) ORO in 60% isopropanol). The stain was then removed, and the cells were washed twice with PBS and photographed.

### Retroviral vectors and infection

To construct a retroviral vector for JMJD1A (pMXs-hJMJD1A-V5-IRES/Zeo), we subcloned DNA sequence encoding hJMJD1A open reading frame into an expression vector pMX (a kind gift from Dr. Toshio Kitamura, The University of Tokyo) driven by LTR promoter, in which the original puromycin-resistant sequences were replaced by Zeocin^TM^-resistant sequences^13^. Mutant versions of hJMJD1A: S265A, S265D, or S265D-H1120Y were generated by PCR-based site-directed mutagenesis. To construct retroviral vector for shRNA targeting Jmjd1a, forward primer: 5′-gatctttgccgatgacctttcagataattcaagagattatctgaaaggtcatcggttttgttcc-3′ and reverse primer: 5′-agctggaacaaaaccgatgacctttcagataatctcttgaattatctgaaaggtcatcggcaaa-3′ were annealed and subcloned into a mouse U6 promoter-driven expression vector pRetro/Puro-Super3 (a kind gift from Dr. T. Kitamura), in which puromycin-resistant marker sequences were replaced by neomycin-resistant marker sequences. Retroviruses were produced by transfecting each plasmid into Platinum-E packaging cells (a kind gift from Dr. T. Kitamura), using GeneJuice (Novagen). To establish JMJD1A knockdown immortalized adipocytes, im-scWATs were transduced with retroviral vector expressing shRNA targeting murine *Jmjd1a* under the control of U6 promoter, and were selected by G418 (1 mg ml^−1^) for 6 days. To establish im-scWATs ectopically expressing WT or mutants JMJD1A, the resultant JMJD1A knockdown im-scWATs were infected with retrovirus-expressing WT, S265A, S265D, or S265D-H1120Y, and the infected cells were selected by zeocin treatment (0.1 mg ml^−1^) for 12 days^13,36^.

### Co-imunoprecipitation and immunoblot analysis

Whole-cell lysates were collected in cell lysis buffer A (50 mM HEPES-KOH, pH 7.9, 150 mM NaCl, 1.5 mM MgCl_2_, and 1% NP-40), containing both protease inhibitors (5 μg ml^−1^ pepstatin A, 10 μg ml^−1^ leupeptin, 2.8 μg  ml^−1^ aprotinin, 1 mM dithiothreitol and 0.5 mM phenylmethylsulfonyl fluoride) and phosphatase inhibitors (40 mM NaF and 1 mM Na_3_VO_4_). JMJD1A or PRDM16 protein was immunoprecipitated in cell lysis buffer A for 6 h at 4 °C using the antibodies described in the legend to figures. Co-immunoprecipitates were subjected to sodium dodecyl sulfate-polyacrylamide gel electrophoresis (SDS-PAGE), and immunoblots were visualized by chemiluminescence using Super Signal West Dura Extended Duration Substrate (Thermo Fisher Scientific), and luminescent images were analyzed by ImageQuant LAS 4000mini (GE Healthcare)^13,36^. For immunoblot analysis of histones, histones were acid extracted from BAT or scWAT of C57BL/6J mice using a Histone Purification Mini Kit (Active Motif), following the manufacturer’s instructions as described^37^.

### ChIP and ChIP-seq

The procedures for H3K9me2 ChIP assay using adipose tissues of mice were performed as follows. Fresh scWATs or BATs were minced, cross-linked with 0.5% (vol vol^−1^) formaldehyde for 10 min, homogenized with Dounce-type homogenizer in ice-cold hypotonic buffer B (10 mM HEPES-KOH, pH 7.5, 1.5 mM MgCl_2_, 10 mM KCl, 1 mM EDTA, 1 mM EGTA containing 1 mM phenylmethylsulfonyl fluoride (PMSF) and protease inhibitor cocktail (Nacalai Tesque)), and centrifuged at 1000 × *g* for 7 min to obtain a nuclear fraction. Cross-linked nuclear fractions were lysed in cell lysis buffer C (23 mM Tris-HCl, pH 8.0, 3.0 mM EDTA, 0.9% Triton X-100, 134 mM NaCl, 1 mM PMSF, and protease inhibitor cocktail (Nacalai Tesque)) containing 0.2% SDS (Fig. 1c and Supplementary Fig. 1c) or 0.1% SDS (Figs. 1f, g and 5d, h, j), sheared from 200 to 300 bp by using SONIFIER 250 (Branson) (output 4, duty cycle 60%, 20 s x 15 times), and used for immunoprecipitation. The amount of DNA content in the nuclear lysate was determined after pronase protein digestion and DNA purification. For H3K9me2 ChIP, 3 μg nuclear DNA containing nuclear lysates were immunoprecipitated for 2 h at 4 °C with 2 μg of anti-H3K9me2 antibody (IgG-6D11)^38^ pre-bound to 50 μl of Dynabeads protein G (Life Technologies). The immunoprecipitated DNA was subjected to qPCR with SYBR green fluorescent dye^13,36^. H3K9me2 ChIP using mouse scWAT ChIP in cell lysis buffer C containing 0.1% SDS (Figs. 1f and 5d) gave higher amount of precipitated DNA, compared to that in cell lysis buffer C containing 0.2% SDS (Fig. 1c).

The procedures for H3K9me2 and PPARγ ChIP assays using culture cells were described previously^13,36^. Briefly, cells cross-linked with 0.5% (vol vol^−1^) formaldehyde for 10 min were homogenized by passing through a 22 G needle 10 times in ice-cold hypotonic buffer B. Relative ChIP enrichment in Fig. 5j represents the ratios of percentage input in the cells differentiated for indicated time, compared with in undifferentiated cells, which are defined as 1.

JMJD1A ChIP for scWATs was performed as follows. Fresh scWATs were minced, cross-linked with 1.5 mM ethylene glycol bis(succinimidylsuccinate) (Thermo Scientific) for 30 min at room temperature (RT), and then directly a second crosslinking was performed by the addition of 1% formaldehyde for 10 min. Nuclear pellets were resuspended in 2 ml cell lysis buffer C containing 0.2% SDS, and chromatin DNA was sheared to 2 kb average in size through sonication. For ChIP, 5 μg nuclear DNA containing nuclear lysates harvested from 140 to 200 mg scWAT per head were immunoprecipitated overnight at 4 °C with a mixture of two anti-JMJD1A antibodies (20 μg each of IgG-F0618 and IgG-F0231) pre-bound to 200 μl of Dynabeads protein G.

For JMJD1A ChIP in cultured cells, we used 30 μg nuclear DNA containing cross-linked nuclear lysates. For ChIP-seq analysis using JMJD1A antibody, precipitated DNA from original DNA size of ~2 kb was further sheared to ~200 bp by an Acoustic Solubilizer (Covaris). For ChIP-seq using PPARγ antibody, DNA sonicated to be 0.5 kb average in size was used for ChIP. Chromatin immunoprecipitated samples were used for library preparation with KAPA Hyper Prep Kit (Nippon Genetics), according to the manufacturer’s instructions^13,36^. Deep sequencing was performed on a HiSeq 2500 sequencer (Illumina) as single-end 50 base reads. All bound DNA fragments were mapped to UCSC build mm9 (NCBI Build 37) assembly of the mouse genome by a mapping program ELAND (Illumina), based on the 36 bp sequences. Signals of ChIP-seq occupancy are presented as reads per million mapped reads (RPM).

### Quantitative real-time PCR

qPCR was carried out in 384-well plates using the ABI PRISM 7900HT sequence detection system (Applied Biosystems). All reactions were done in triplicate. All primer sequences used in this article are listed and shown in the Supplementary Tables 3 and 4. Copy numbers of *Ucp1* mRNA in BAT and scWAT were determined by qPCR, as described^37^, using a plasmid containing mouse *Ucp1* open reading frame as the standard template for *Ucp1*. The data were converted to copy number per ng of the total RNA.

### RNA-sequencing

Total RNA of the cells was isolated using ISOGEN (Nippon Gene), according to the manufacturer’s protocol. RNA-Seq libraries were prepared by TruSeq Sample Purification Kit (Illumina). The libraries were sequenced on HiSeq 2500 (Illumina), and the reads were aligned to mouse transcriptome (UCSC gene) and genome (mm9) references, respectively, using Burrows-Wheeler aligner. After transcript coordinates were converted to genomic positions, an optimal mapping result was selected either from the transcript or from the genome mapping by comparing the minimal edit distance to the reference. Local realignment was performed within in-house short reads aligner with smaller *k*-mer size (*k* = 11). Finally, FPKM values were calculated for each UCSC gene, while considering strand-specific information. When expression alterations were analyzed, gene expression levels are presented as log_2_ fold change in FPKM, relative to those in indicated controls.

### Analysis of transcription factor-binding motifs

Transcription factor-binding motif analysis was performed using TRAP, in which the algorithm ranks the known binding motifs in descending order of the enrichment in target genomic sites^39^. The JMJD1A binding sites^13^ from ChIP-seq data were extracted by SICER using the default parameter setting: window size, 200 bp; gap size, 400 bp; *E*-value threshold, 100. Subsequently, the PPARγ binding sites^13^ and the PRDM16 binding sites^26^ from the ChIP-seq data were extracted by MACS using the default parameter setting: *p* value threshold, 5e−10; band width, 300 bp; shift size, 100 bp. The sites co-localized by JMJD1A, PPARγ, and PRDM16, and those localized by only JMJD1A were, respectively, obtained by comparing the overlaps among the factor bindings. In the two datasets, after the sizes of all the binding sites were converted to 500 bp, the sites with top-300 JMJD1A binding signals were selected as the input data of TRAP. After that, the enriched binding motifs were predicted and were compared with random promoters. As the reference motifs, we used those archived in JASPAR, the database of the known binding motif. The top-5 significant binging motifs were displayed in Supplementary Figure 4f.

### Measurements of oxygen consumption

Oxygen consumption was measured using a Seahorse XF24 extracellular flux analyzer, as described previously^13^. Briefly, the cultured adipocytes at day 7 of the differentiation were detached with Trypsin (0.5 g l^−1^)/EDTA (0.53 mM) solution (Nacalai Tesque) and re-seeded at 5.0 × 10^4^ or 1.0 × 10^5^ cells per well for brown adipocytes and white or beige adipocytes, respectively, into XF24 V7 cell culture microplates (Seahorse Bioscience). First, the basal respiration was assessed in untreated cells. Second, ATP turnover was calculated in response to 40 μg ml^−1^ oligomycin (Oligo) (Wako). Third, maximum respiratory capacity was assessed after the stimulation by 2.5 μM carbonyl cyanide 4-(trifluoromethoxy) phenylhydrazone (FCCP) (Sigma). Finally, mitochondrial respiration was blocked by adding both 1 μM rotenone (Sigma) and 1 μM antimycin A (Wako), and the residual OCR was considered as nonmitochondrial respiration. Proton leak was calculated by subtracting the ATP turnover and the nonmitochondrial respiration components of basal respiration, as described previously^13^. To assess the oxygen consumption of the mouse scWAT, the adipose tissues (1.5 mg) from mice housed at 30 °C or 4 °C for 1 week were isolated, minced, and placed into XF24 Islet Capture Microplates (Seahorse Bioscience), and the basal OCR was monitored after preincubation for 1 h with the XF24 assay media.

### Glycerol release

NE-stimulated glycerol release from differentiated adipocytes from cultured differentiated adipocytes (i.e., hJMJD1A-transduced immortalized adipocytes or SVF of BAT or scWAT) was measured using a Lipolysis Assay Kit (ZenBio), as described^13^. Differentiated adipocytes on day 7 were detached with Trypsin (0.5 g l^−1^)/EDTA (0.53 mM) solution (Nacalai Tesque), and re-seeded at 2.5 × 10^5^ and 1.5 × 10^5^ cells for brown and white adipocytes, respectively, into 96-well plate. The next day (day 8), the cells were treated with NE (10 μM) for 1 h at 37 °C. The optical density of produced quinoneimine dye was measured at 540 nm.

### Mitochondria quantity analyses

For quantification of mitochondrial DNA (mt-DNA) copy number, mt-DNA was isolated from cultured adipocyte by phenol/chloroform, after digestion with proteinase K (100 μg ml^−1^) at 37 °C overnight. Relative amounts of nuclear DNA and mt-DNA were determined by quantitative real-time PCR. We selected NADH dehydrogenase 1, 2, or 4 coding gene (*Nd1*, *Nd2*, or *Nd4*, respectively) and *Cyclophilin* (*Ppib*) for nuclear DNA quantification. For MitoTracker staining, cells were incubated with 100 nM MitoTracker Green FM dyes (Molecular Probes) for 30 min at 37 °C. After staining, the cells were washed with pre-warmed PBS. Immunofluorescence was captured with Leica DMI 6000B microscope (Leica microsystems).

### Immunohistochemistry

BAT and scWAT isolated from mice were placed in 10% formalin for 48 h, stored in 70% ethanol, and subsequently paraffin embedded. Immunostaining was performed on deparaffinized 3 μm sections, which were rehydrated and quenched the endogenous peroxidase activity by treatment with 3% hydrogen peroxide for 15 min. Following antigen epitope retrieval performed by autoclaving the slides in Tris buffer (pH 9.0), the sections were incubated overnight at room temperature with mouse monoclonal anti-UCP1 antibody (ab10983, Abcam), at a dilution of 1:1000. The bound anti-UCP1 was detected by rabbit anti-mouse secondary antibody, followed by biotin-free horseradish peroxidase-labeled polymers (Dako). Bound horseradish peroxidase-labeled polymers were detected by the addition of 3,3-diaminobenzidine substrate-chromogen solution, and counterstained with hematoxylin.

### Electron microscopy analysis

Cell pellets were fixed in 2.5% glutaraldehyde in 0.1 M phosphate buffer (pH 7.4). Post-fixation was performed in 1% osmium tetroxide (OsO_4_) in 0.1 M phosphate buffer (pH 7.4). Subsequently, the samples were dehydrated and embedded in epoxy resin (*Quetol-651*, Nissin EM). Ultrathin sections (100 nm) were cut by an ultramicrotome (Ultracut N, Reichert-Nissei) and collected on grid meshes. The sections were stained with 1% uranyl acetate for 10 min, followed by 1% lead citrate for 5 min, and washed with distilled water. They were examined with an electron microscope (Hitachi H-600A, Hitachi).

### Statistical analysis

All data are presented as mean ± s.e.m. Student’s *t* test was performed for the comparison of two groups. For multiple comparisons, analysis of variance were performed, followed by Tukey’s post hoc comparison. *P < 0.05, **P < 0.01, and ***P < 0.005. Significance was considered as *P* < 0.05.

### Data availability

RNA-seq transcriptome data and ChIP-seq data reported in this paper is deposited to Gene Expression Omnibus in the accession number of GSE107901.

## Electronic supplementary material

Supplementary Information
