# Supplementary material for: Histone demethylase JMJD1A coordinates acute and chronic adaptation to cold stress via thermogenic phospho-switch
Source: Nat Commun. 2018 Apr 19;9:1566. doi: 10.1038/s41467-018-03868-8 (PMC5908789; doi:10.1038/s41467-018-03868-8)
Supplement: Supplementary file 1 — Supplementary Information [file 41467_2018_3868_MOESM1_ESM.pdf]

Histone demethylase JMJD1A coordinates acute and chronic adaptation to cold stress via thermogenic phospho-switch

Abe et al.

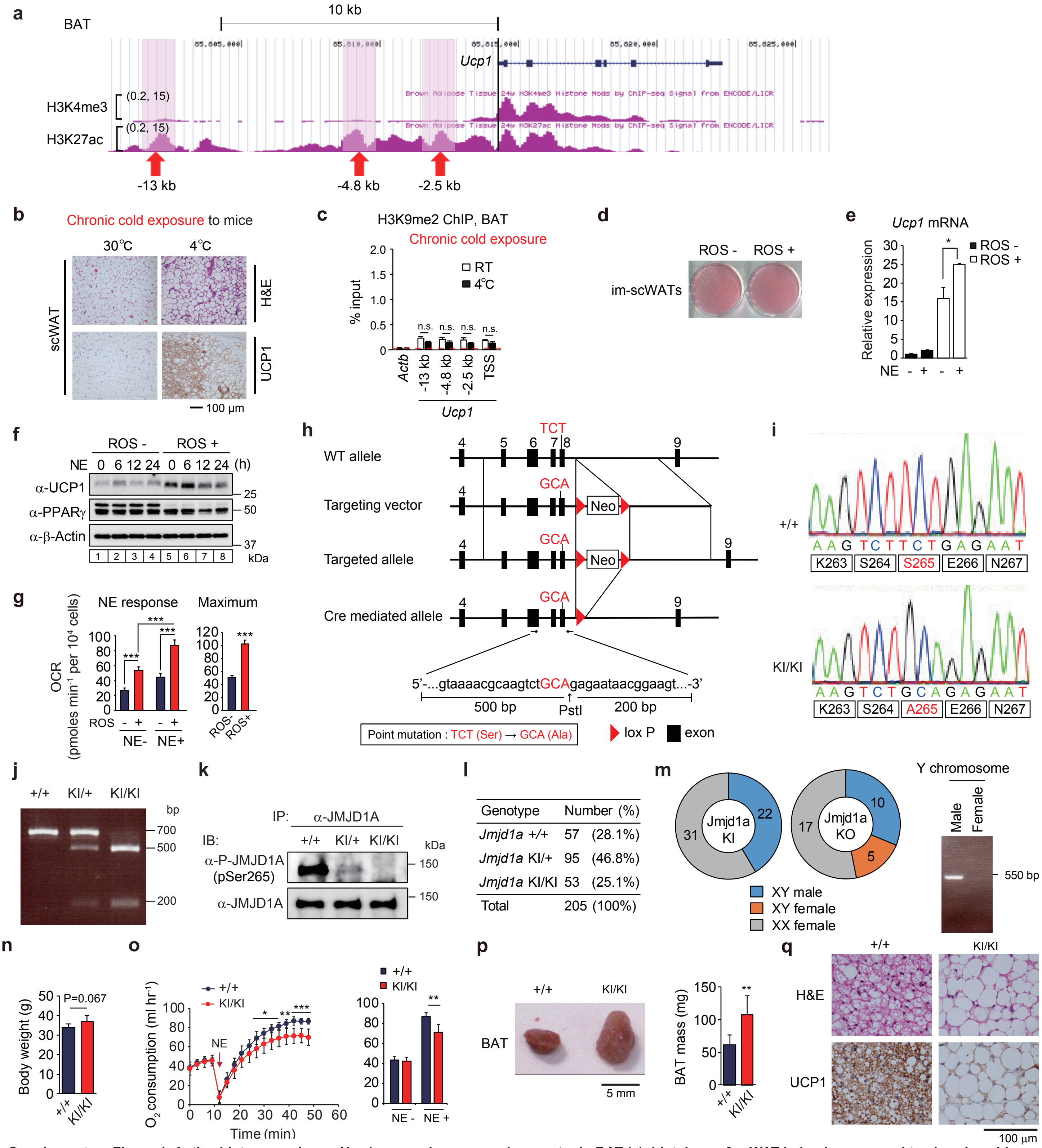

Supplementary Figure 1 Active histone marks on *Ucp1* gene enhancers and promoter in BAT (a), histology of scWAT beige-ing exposed to chronic cold exposure (b), H3K9me2 levels on the enhancers/promoter of *Ucp1* gene in BAT of mice following chronic cold exposure (c), characterization of immortalized scWAT of mice (d-g), and generation of *Jmjd1a*-S265A$^{KI/KI}$ mice (h-q).

(**a**) ChIP-seq profiles of H3K4me3 and H3K27ac on *Ucp1* genomic region in mouse BAT were obtained from ENCODE/LICR histone modification data in the UCSC genome browser (NCBI37/mm9 assembly). Light pink shadows highlight the enhancers as we reported previously (Abe et al. 2015). Scale bar, 10kb. (**b**) Hematoxylin and eosin (H&E) and UCP1 staining sections of scWATs from mice exposed to 30ºC or 4ºC for 1 week (bar, 100 μm). (**c**) ChIP-qPCR showing H3K9me2 levels in BAT of mice exposed to RT or 4ºC for 1 week (7-8 week old male, n = 6). (**d**) Oil red O (ORO) staining in im-scWATs differentiated with or without rosiglitazone (ROS). (**e**) mRNA levels of *Ucp1* in im-scWATs differentiated with or without rosiglitazone (ROS) treated with or without norepinephrine (NE, 10 μM) were measured by qPCR. The mRNA values are depicted relative to mRNA in im-scWATs differentiated without ROS treated without NE, which are arbitrarily defined as 1 (mean ± s.e.m. of three technical replicates). (**f**) Immunoblot analysis of UCP1 and PPARγ in the time course of norepinephrine treatment (NE, 10 μM) in im-scWATs differentiated with or without rosiglitazone (ROS). Equal loading of the proteins was confirmed by β-actin. (**g**) The metabolic profile of im-scWATs differentiated with or without rosiglitazone (ROS) was assessed using a Seahorse XF24 extracellular flax analyzer. The parameters analyzed on the same plate are represented as norepinephrine (NE, 10 μM)-induced mitochondrial (left panel) and maximum mitochondrial respiration (right panel) (mean ± s.e.m. of five technical replicates). (**h**) Schematic diagram of the targeting strategy of S265A mutation. Only the relevant restriction sites are indicated. Locations of the PCR primers (arrows) for genotyping are shown. (**i**) Direct sequencing of genomic DNA from mice distinguishes between *WT* (+/+) mice (Serine 265: TCT) and *Jmjd1a*-S265A$^{KI/KI}$ mice (Alanine 265: GCA). (**j**) An ethidium bromide-stained agarose gel illustrates PCR products for genotyping *WT*, *Jmjd1a*-S265A$^{KI/+}$ mice, and *Jmjd1a*-S265A$^{KI/KI}$ mice. (**k**) Subcutaneous white adipose tissues (scWATs) were isolated from *WT* and *Jmjd1a*-S265A$^{KI/KI}$ male mice housed at 4ºC for 3 hr. Tissue homogenate from *WT* scWAT and *Jmjd1a*-S265A$^{KI/KI}$ scWAT were subjected to immunoprecipitation (IP) with anti-mJMJD1A followed by immunoblot (IB) analysis with anti-P-JMJD1A (pSer265). Uncropped images of the blots (**f**,**k**) are shown in **Supplementary Fig. 8**. (**l**) Genotype in pups (n = 205) obtained by crossing *Jmjd1a*-S265A$^{KI/+}$ mice. (**m**) Quantification of XY male, XY female, and XX female (left panel). An ethidium bromide-stained agarose gel illustrates PCR product for identifying sex (right panel). The PCR product (550 bp) by forward primer 5'-AAGATAAGCT-TACATAATCACATGGA-3' and reverse primer 5'-CCTATGAAATCCTTTGCTGCACATGT-3' was detected in male (Y chromosome). (**n**) Body weights of *WT* (+/+) and *Jmjd1a*-S265A$^{KI/KI}$ male mice (n = 6/group). (**o**) Norepinephrine (NE, 1 mg/kg BW)-induced changes in O_2 consumption of *WT* and *Jmjd1a*-S265A$^{KI/KI}$ mice acclimated to 30ºC for 2 weeks (left). O_2 consumption rates before and 30 min after NE treatment were analyzed (right) (n = 6/group). (**p**) Representative images (bar, 5 mm) (left) and the weights (right) of BAT of *WT* (+/+) and *Jmjd1a*-S265A$^{KI/KI}$ mice described in (**n**) (n = 6/group). (**q**) Haematoxylin and eosin (H&E) and UCP1 staining sections of BAT from *WT* and *Jmjd1a*-S265A$^{KI/KI}$ mice (bar, 100 μm). (**c**,**e**,**g**,**n-p**) Data are mean ± s.e.m. Student's t-test was performed for comparisons in **c**,**e**,**g**, right panel; **n-p** and analysis of variance were performed followed by Tukey's post hoc comparison in **g**, left panel. *P < 0.05, **P < 0.01 and ***P < 0.005 were considered statistically significant. n.s. not significant.

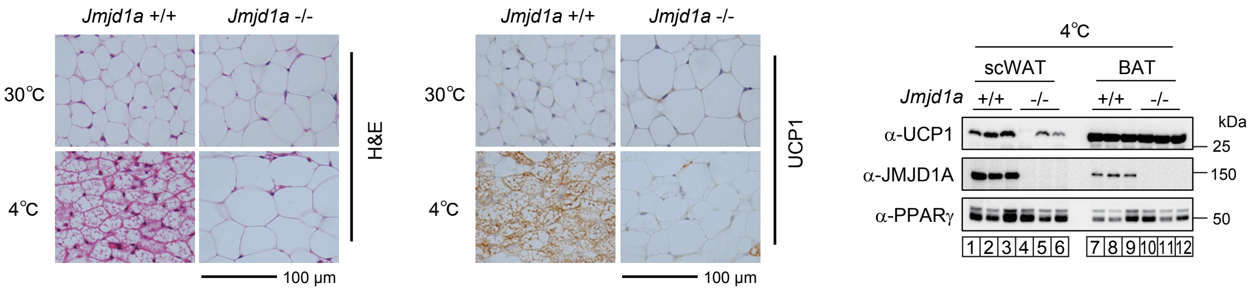

Supplementary Figure 2 Impaired beige-ing of scWAT in *Jmjd1a*-null mice by chronic cold exposure.
Hematoxylin and eosin (H&E) and UCP1 staining sections of scWATs from *WT* (+/+) and *Jmjd1a*-null (-/-) mice exposed to 30ºC or 4ºC for 1 week (left and middle panels) (bar, 100 μm). Tissue homogenate of scWATs and BAT from these mice exposed to 4ºC were subjected to immunoblot analysis with anti-UCP1, anti-JMJD1A, or anti-PPARγ (right). Uncropped images of the blots are shown in **Supplementary Fig. 8**.

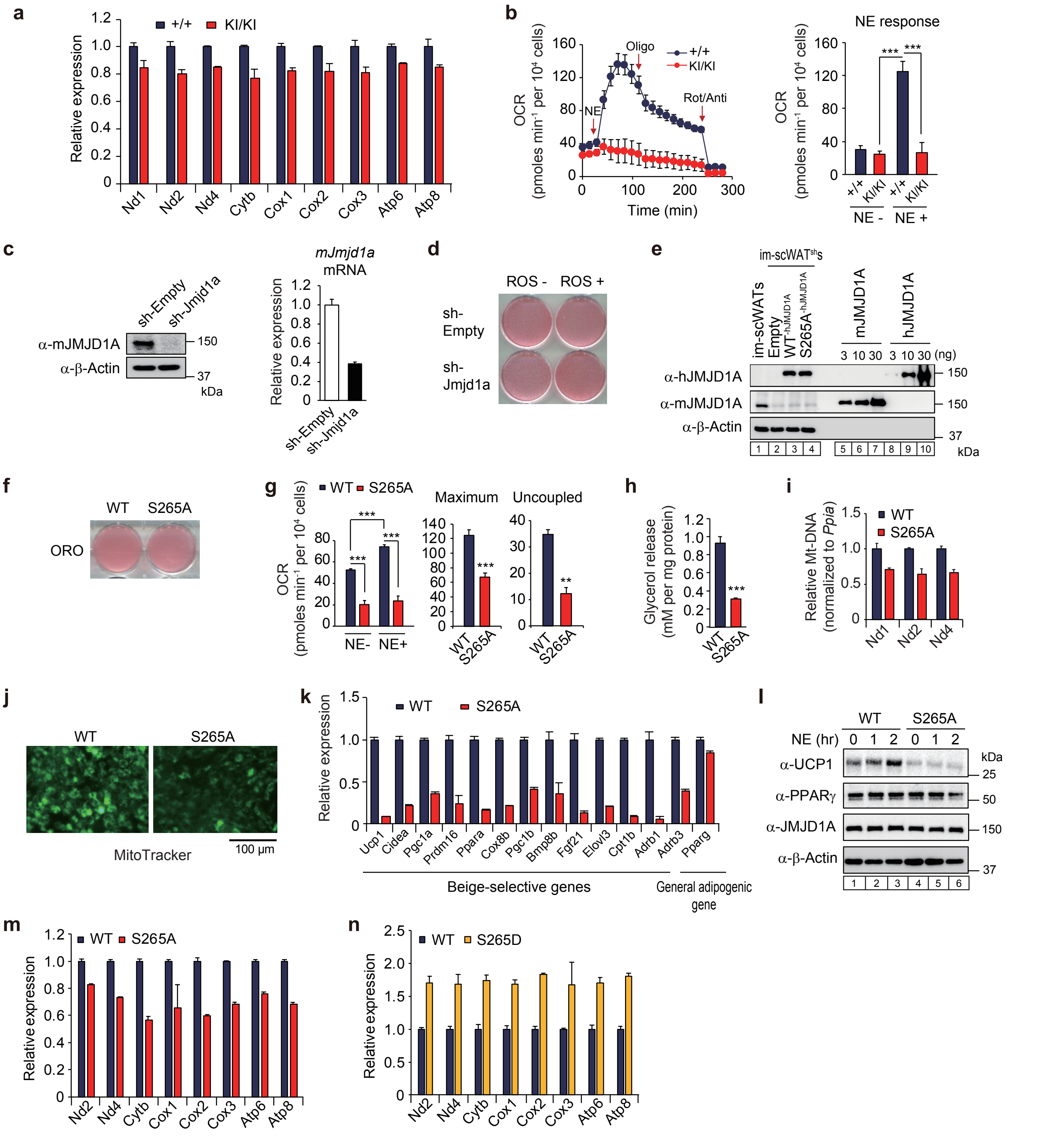

Supplementary Figure 3 pS265-JMJD1A cell autonomously induces beige adipogenesis.

(**a**,**b**) Stromal vascular fractions (SVF) from scWAT of *WT* or *Jmjd1a*-S265A$^{KI/KI}$ were cultured and induced for beige adipogenesis. (**a**) mRNA levels measured by qPCR. The mRNA values are depicted relative to mRNA in *WT* culture, which are arbitrarily defined as 1 (mean ± s.e.m. of three technical replicates). (**b**) The metabolic profile assessed by Seahorse XF24 extracellular flux analyzer (left). The parameters analyzed on the same plate are represented as norepinephrine (NE, 10 μM)-induced mitochondrial respiration (right) (mean ± s.e.m. of five technical replicates). The arrows indicate time of addition for oligomycin (Oligo), FCCP, and rotenone/antimycin A (Rot/Anti). (**c**) Immunoblot (left) and qPCR analysis (right). The mRNA values are depicted relative to mRNA in im-scWATs (sh-Empty), which are arbitrarily defined as 1. Data are mean ± s.e.m. of three technical replicates. (**d**) Oil red O (ORO) staining in im-scWATs expressing shRNA targeting mouse *Jmjd1a* (sh-Jmjd1a) or empty vector (sh-Empty) treated with or without rosiglitazone (ROS). (**e**) Exogenous human JMJD1A expression level in im-scWAT^{sh}s was similar level to native JMJD1A in im-scWATs. Aliquots of whole cell lysate prepared from im-scWATs, im-scWATs knocked-down Jmjd1a by shRNA (im-scWAT^{sh}s) overexpressing WT or S265A human JMJD1A, or control Zeo^{r}-empty (Empty) along with the indicated amounts of recombinant purified human JMJD1A (full length) or His-tagged mouse JMJD1A (full length) proteins were subjected to IB analysis with anti-mJMJD1A antibody or anti-hJMJD1A antibody. (**f**) Oil red O (ORO) staining. (**g**) The metabolic profile of WT- and S265A-^{hJMJD1A}-transduced im-scWATs differentiated with ROS assessed by a Seahorse XF24 extracellular flux analyzer. The parameters analyzed on the same plate are represented as norepinephrine (NE, 10 μM)-induced mitochondrial respiration (left), maximum mitochondrial respiration (middle) and uncoupled mitochondrial respiration (right) (mean ± s.e.m. of five technical replicates). (**h**) Glycerol release in WT- and S265A-^{hJMJD1A}-transduced im-scWATs treated with NE (10 μM) for 2 hr (mean ± s.e.m. of three independent experiments). (**i**) Mitochondrial DNA (Mt-DNA) content. The mt-DNA values are depicted relative to mt-DNA in WT-^{hJMJD1A}-transduced im-scWATs, which are arbitrarily defined as 1 (mean ± s.e.m. of three independent experiments). (**j**) MitoTracker staining in WT- and S265A-^{hJMJD1A}-transduced im-scWATs (bar, 100 μm). (**k**) mRNA levels of beige-selective genes and general adipogenic gene in WT- and S265A-^{hJMJD1A}-transduced im-scWATs differentiated with rosiglitazone were measured by qPCR. The mRNA values are depicted relative to mRNA in WT-^{hJMJD1A}-transduced im-scWATs, which are arbitrarily defined as 1 (mean ± s.e.m. of three technical replicates). (**l**) Immunoblot analysis of UCP1, PPARγ and JMJD1A in the time course of norepinephrine (NE, 10 μM) treatment in WT- and S265A-^{hJMJD1A}-transduced im-scWATs differentiated with rosiglitazone. Equal loading of the proteins was confirmed by β-actin. Uncropped images of the blots (**c**,**e**,**l**) are shown in **Supplementary Fig. 8**. (**m**,**n**) mRNA levels (**m**) or S265D-^{hJMJD1A} (**n**)-transduced im-scWATs differentiated with ROS were measured by qPCR. The mRNA values are depicted relative to mRNA in WT-^{hJMJD1A}-transduced cultures, which are arbitrarily defined as 1 (s.e.m. of three technical replicates). (**b**,**g**,**h**) Student's t-test was performed for comparisons in **g**, middle and right panels; **h** and analysis of variance were performed followed by Tukey's post hoc comparison in **b**, right panel; **g**, left panel. $^{**}P < 0.01$ and $^{***}P < 0.005$ were considered statistically significant.

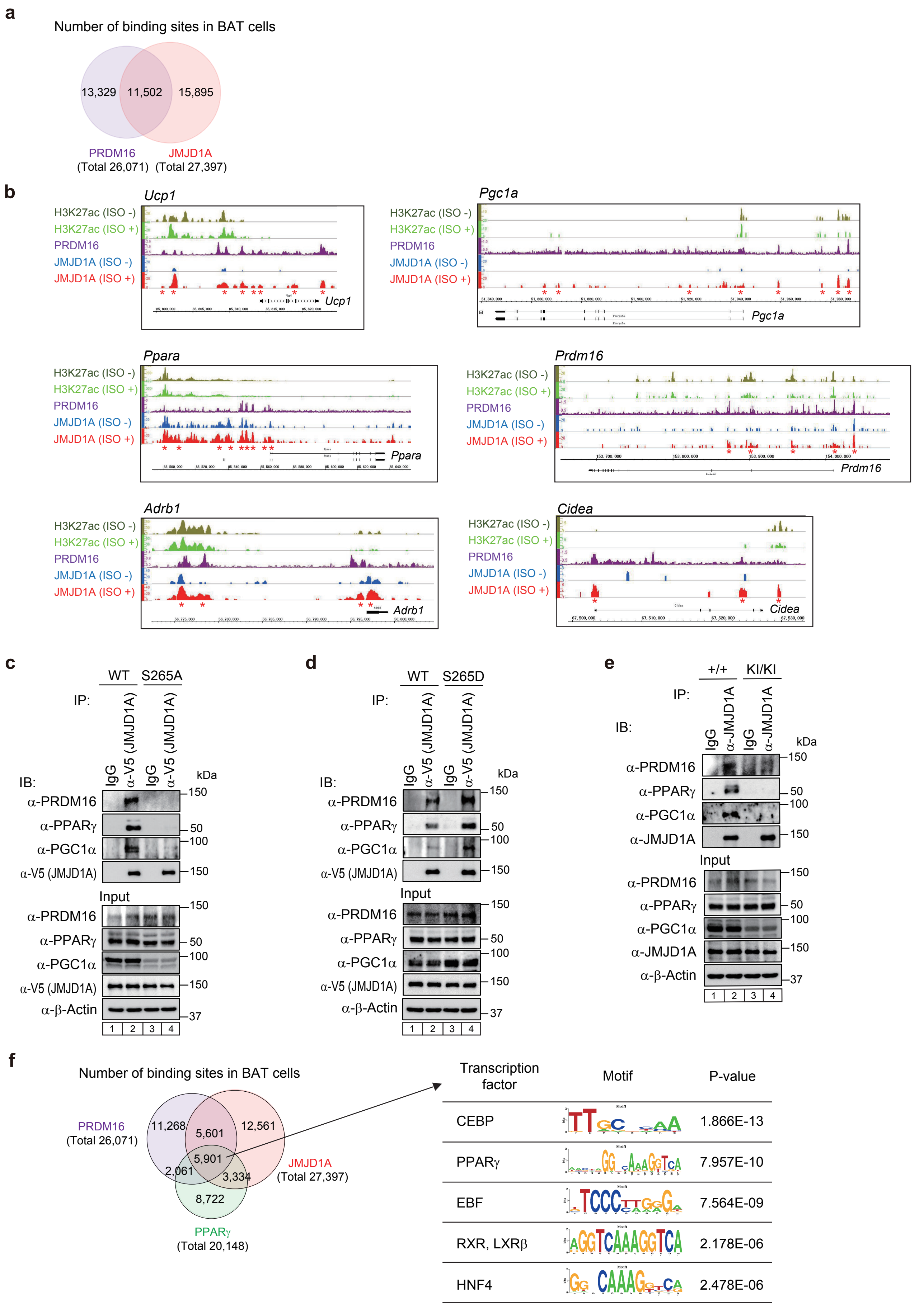

Supplementary Figure 4 β-adrenergic signal induces pS265-JMJD1A-PPARγ-PGC1α-PRDM16 protein complex formation.
(**a**) Venn diagram shows the number of genome-wide DNA binding sites of PRDM16 and JMJD1A in brown adipocytes following the stimulation with β-adrenergic agonist, isoproterenol (ISO). (**b**) ChIP-seq profiles for H3K27ac, PRDM16 and JMJD1A in brown adipocytes treated with or without β-adrenergic agonist, isoproterenol (ISO, 1 μM for 2 hr) on beige-selective genes. Red asterisks indicate ISO-dependent JMJD1A binding sites overlapped with those of PRDM16. (**c**,**d**) Whole-cell lysates (WCL) from WT-, S265A- (**c**) or S265D-hJMJD1A (**d**)-transduced im-scWATs were subjected to immunoprecipitation (IP) with anti-V5 antibody followed by immunoblot (IB) analysis with either anti-PRDM16, PPARγ, PGC1α or V5 antibodies. (**e**) WCL from *WT* (+/+) and *Jmjd1a*-S265A$^{KI/KI}$ scWAT cultures was subjected to immunoprecipitation (IP) using anti-JMJD1A antibody and immunoblotted (IB) with either anti-PRDM16, PPARγ, PGC1α or JMJD1A antibodies. Uncropped images of the blots (**c**,**d**,**e**) are shown in **Supplementary Fig. 8**. (**f**) Venn diagram shows the number of genome-wide DNA binding sites of PRDM16, JMJD1A and PPARγ in brown adipocytes treated with ISO (left panel). Transcription factor binding motifs enriched in genomic regions within JMJD1A, PRDM16 and PPARγ bindings are listed in the right panel. Data for JMJD1A, H3K27ac and PPARγ were obtained from Abe et al., 2015 (Nat Commun 6, 7052 (2015)) and PRDM16 from Harms et al., 2015 (Genes Dev 29, 298-307 (2015)) in **a**, **b**, **f**.

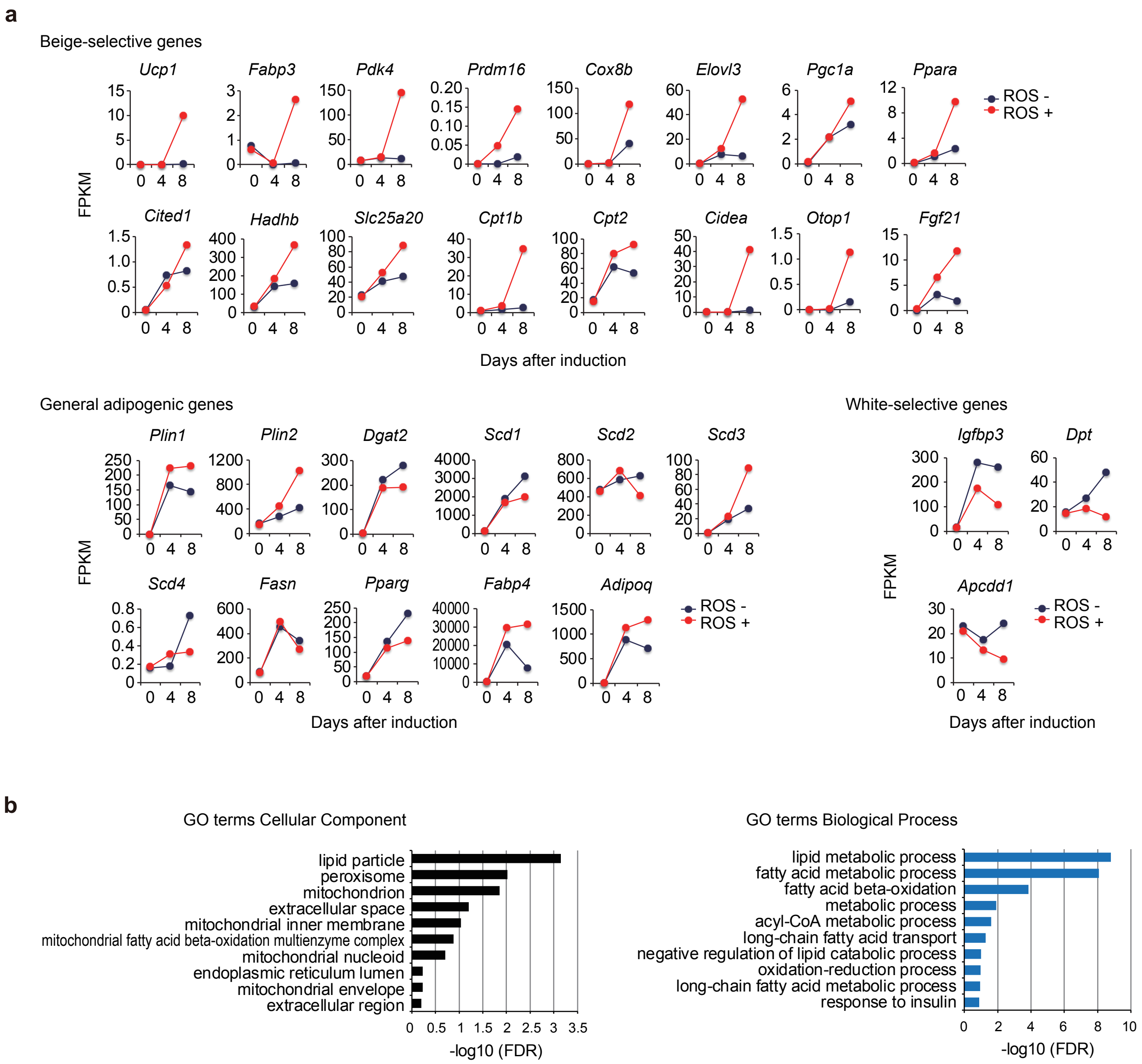

Supplementary Figure 5 Transcriptional changes associated with beige adipogenesis.
(**a**) mRNA levels for beige-selective genes, general adipogenic genes, and white-selective genes in im-scWATs differentiated with or without rosiglitazone (ROS), as determined by RNA-Seq and are expressed as FPKM**.** (**b**) GO analysis of pS265-JMJD1A dependent 126 beige-selective genes described in **Figure 5a**.

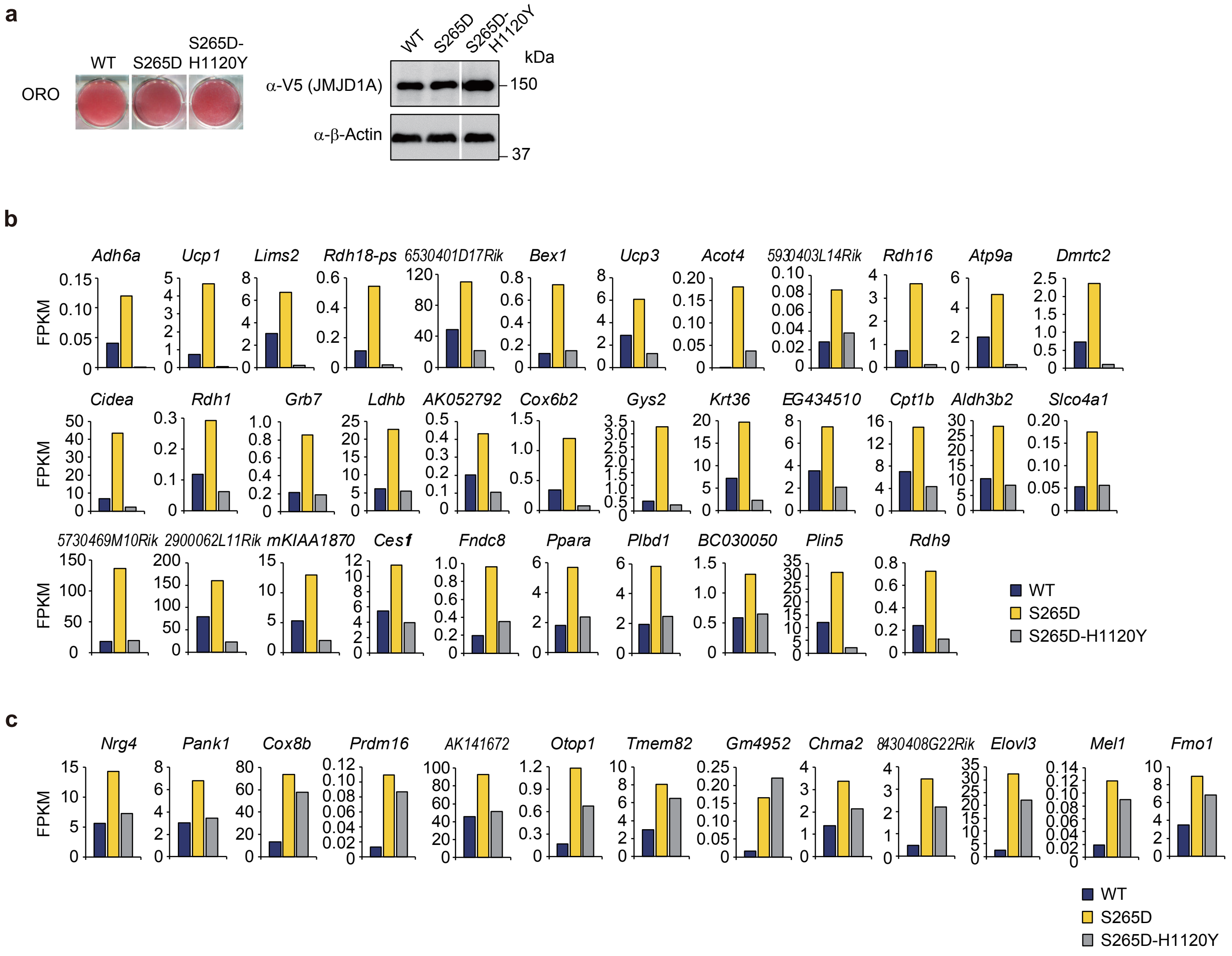

Supplementary Figure 6 Demethylation activity of JMJD1A is pivotal for beige-selective gene inductions.
(**a**) ORO staining in WT-, S265D- or S265D-H1120Y-hJMJD1A-transduced im-scWATs differentiated with rosiglitazone (left panel). Whole-cell lysates from WT-, S265D- S265D-H1120Y-hJMJD1A-transduced im-scWATs differentiated with rosiglitazone were subjected to immunoblot analysis with anti-V5 antibody (right panel). Uncropped images of the blots are shown in **Supplementary Fig. 8**. (**b**,**c**) mRNA levels for 47 genes that are beige-selective, S265A down-regulated, and S265D up-regulated as determined by RNA-Seq and are expressed as FPKM. Top 34 genes (**b**) of 47 genes reduced more than half in mRNA expression from the S265D-H1120Y compared to the S265D. The rest of 13 genes are shown in **c**.

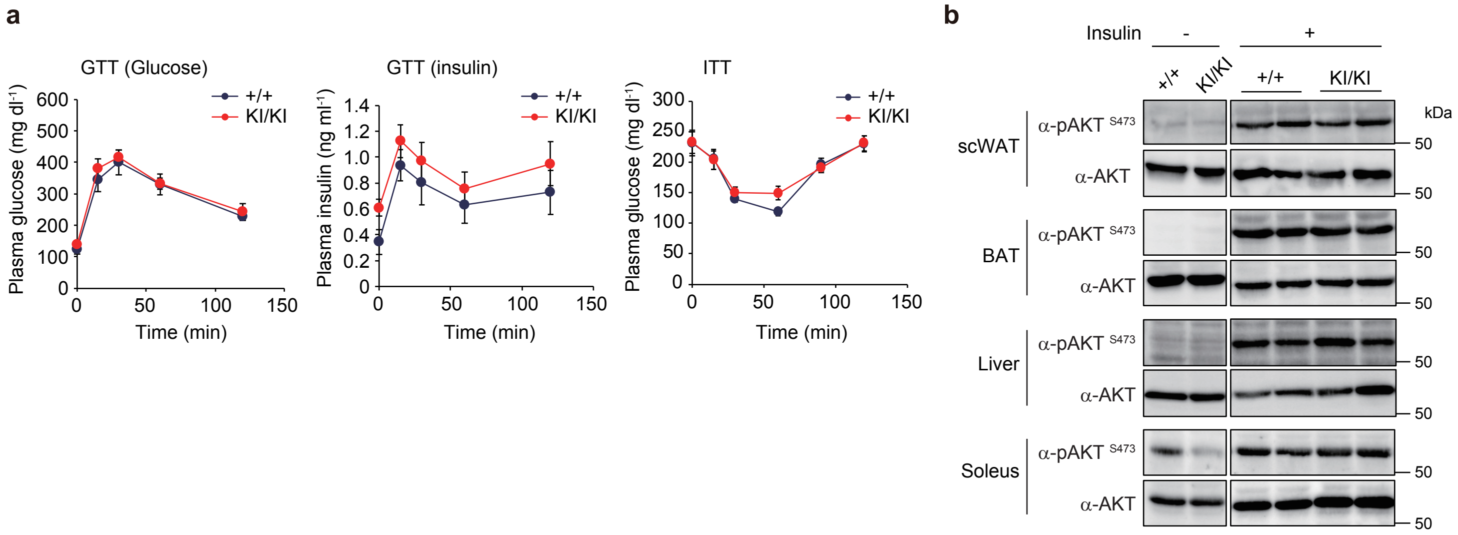

Supplementary Figure 7 Similar glucose tolerance and insulin sensitivity in *Jmjd1a-S*265A$^{KI/KI}$ mice and *WT* mice housed under thermoneutrality.
(**a**) Glucose tolerance test (GTT) and insulin tolerance test (ITT) were performed in *WT* (+/+) and *Jmjd1a*-S265A$^{KI/KI}$ mice fed on a high fat diet (HFD) before cold acclimation (n = 6-7/group). Plasma glucose levels (left panel) and plasma insulin levels (middle panel) during GTT and plasma glucose levels (right panel) during ITT are shown. Data are mean ± s.e.m. (**b**) Assessment of insulin signaling, as quantified by the phosphorylation of AKT-S473, in scWAT, BAT, liver or soleus muscle from *WT* and *Jmjd1a*-S265A$^{KI/K}$ mice housed at room temperature before and 10 min after insulin injection (0.03 unit). Uncropped images of the blots are shown in **Supplementary Fig. 8**.

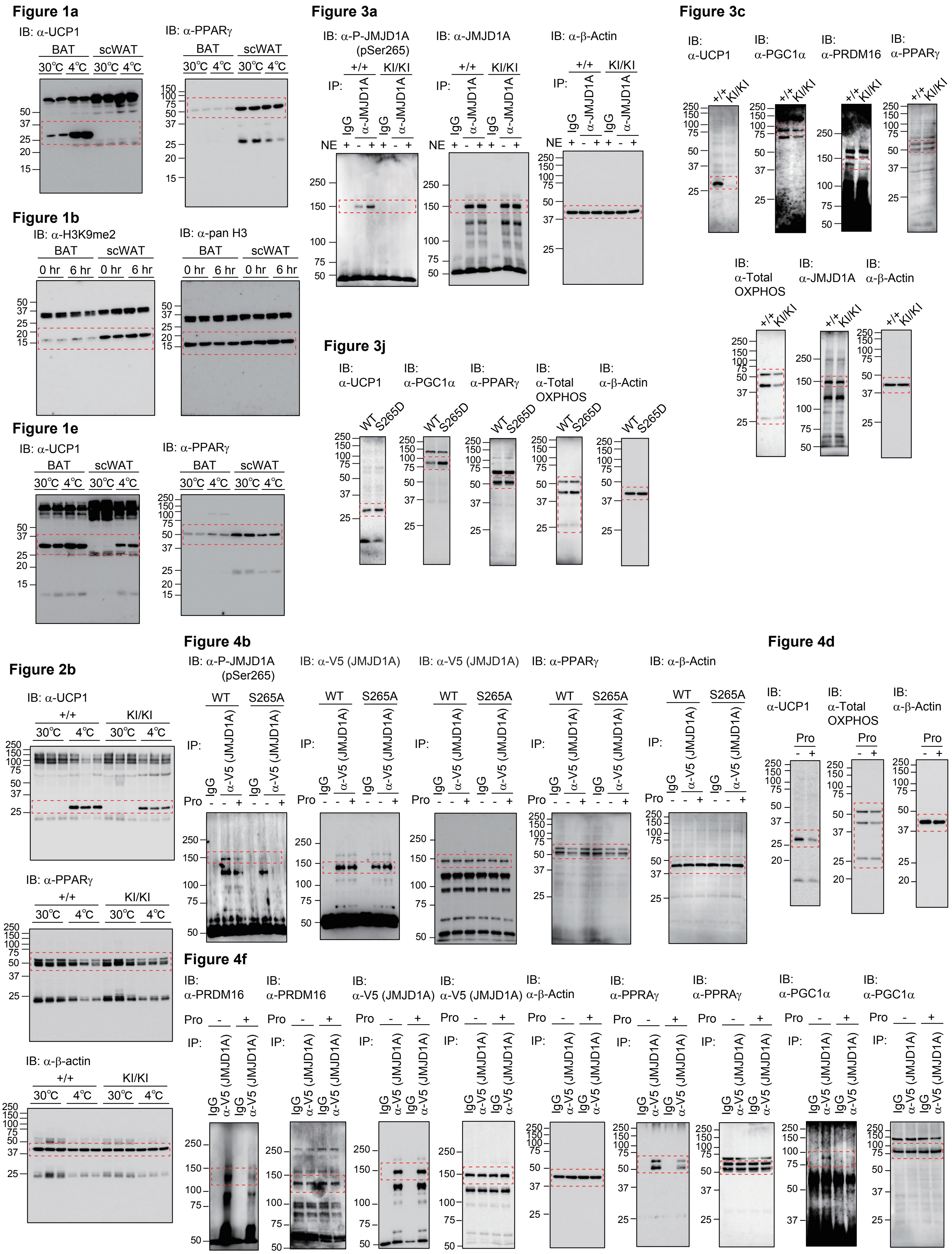

Supplementary Figure 8 Representative original images of immunoblot analysis

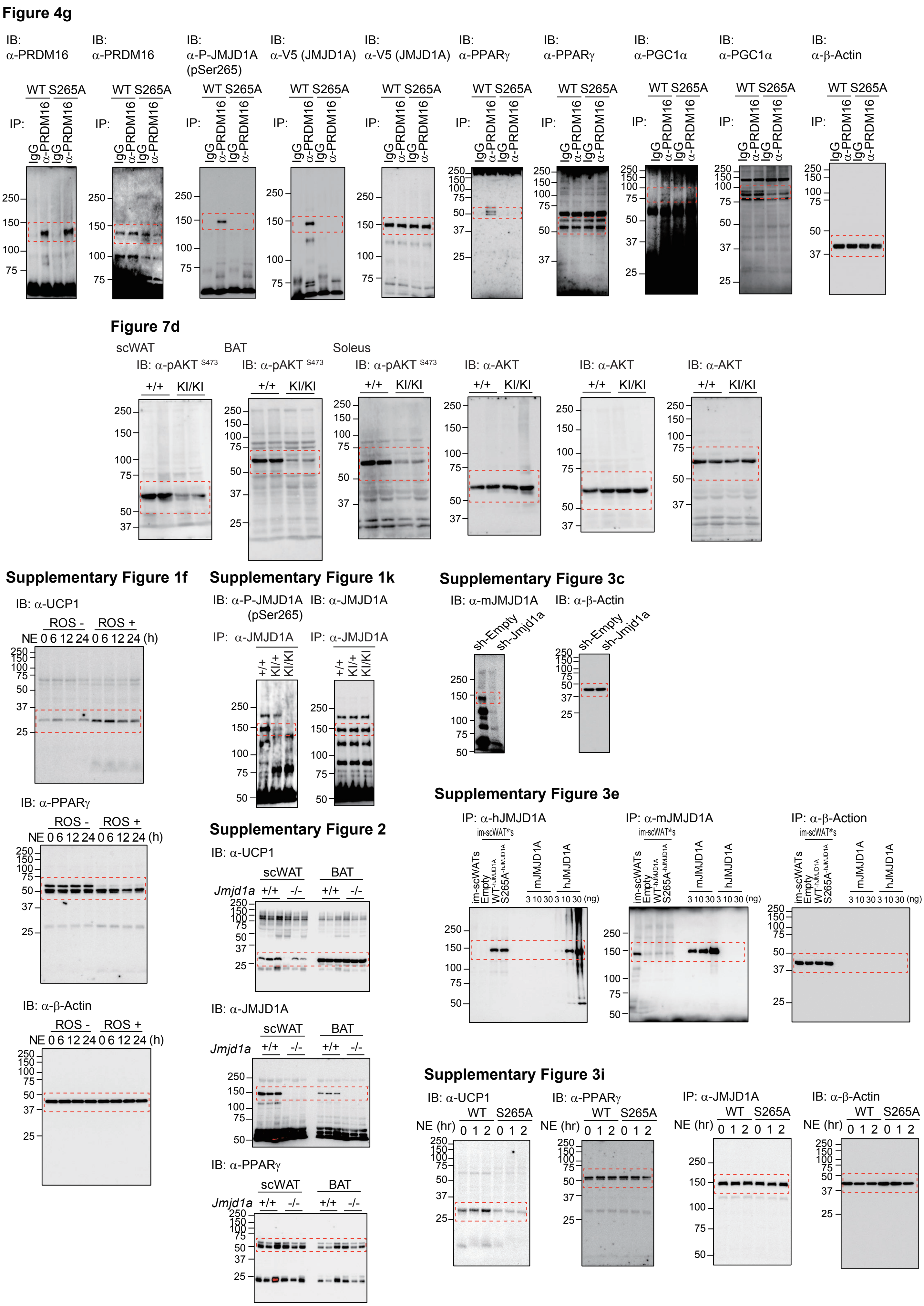

Supplementary Figure 8 Continued

Supplementary Figure 4c

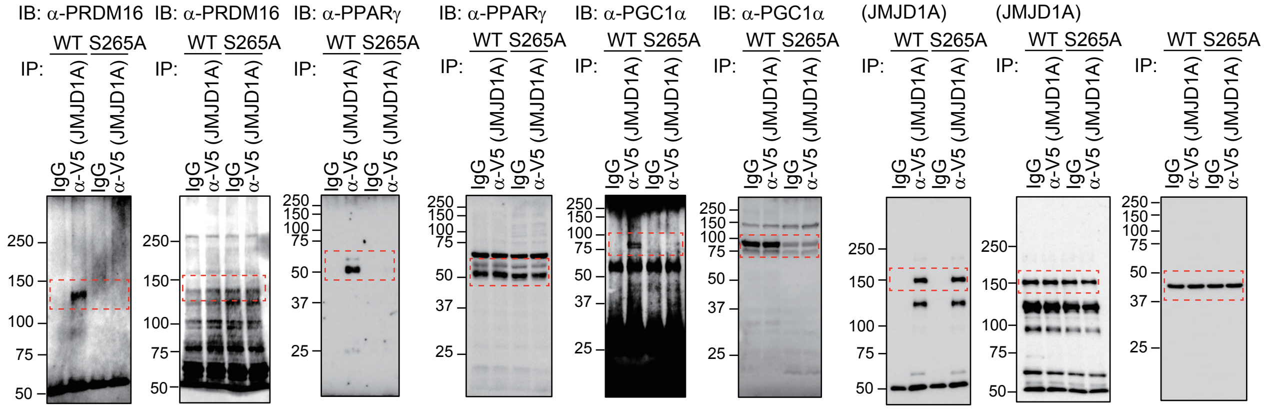

Supplementary Figure 4d

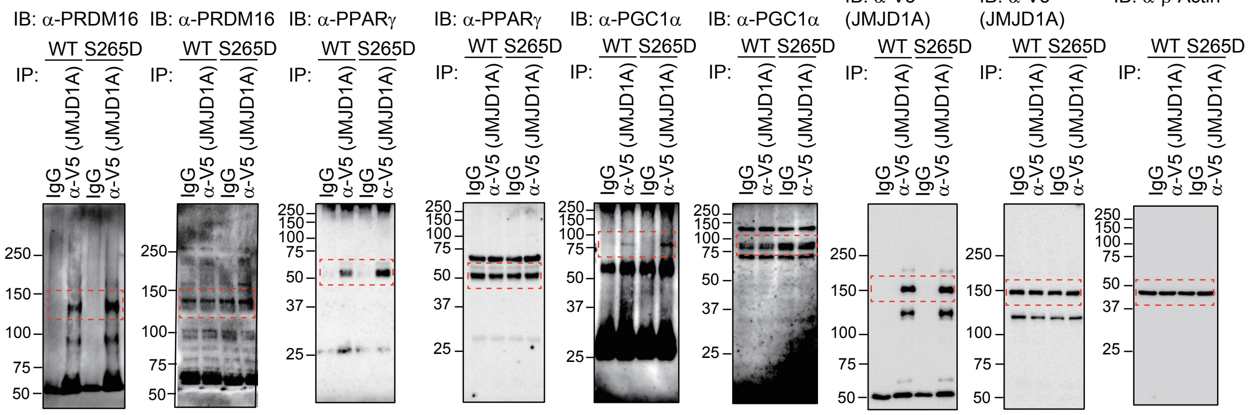

Supplementary Figure 4e

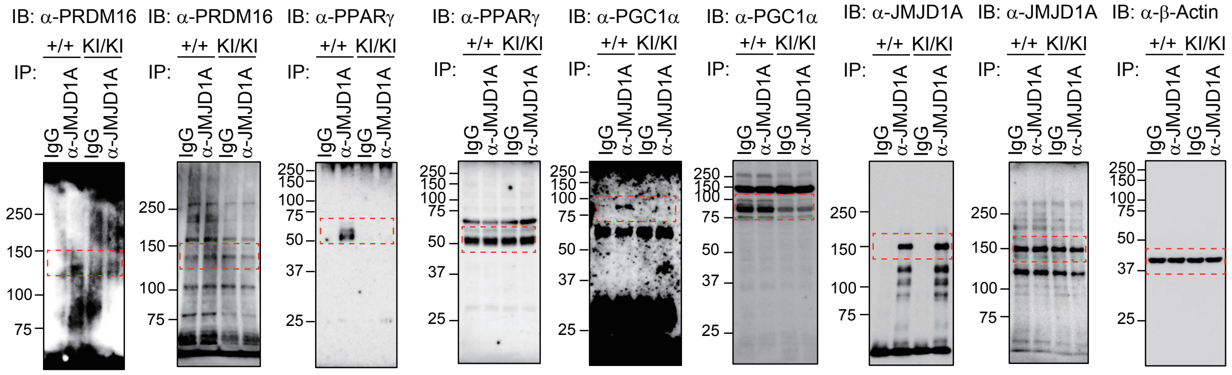

Supplementary Figure 6a

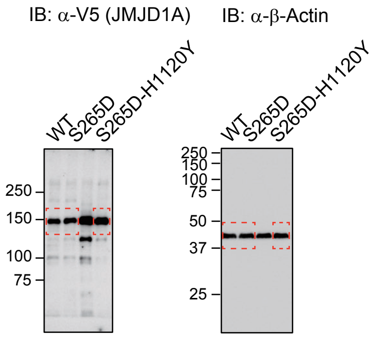

Supplementary Figure 7b

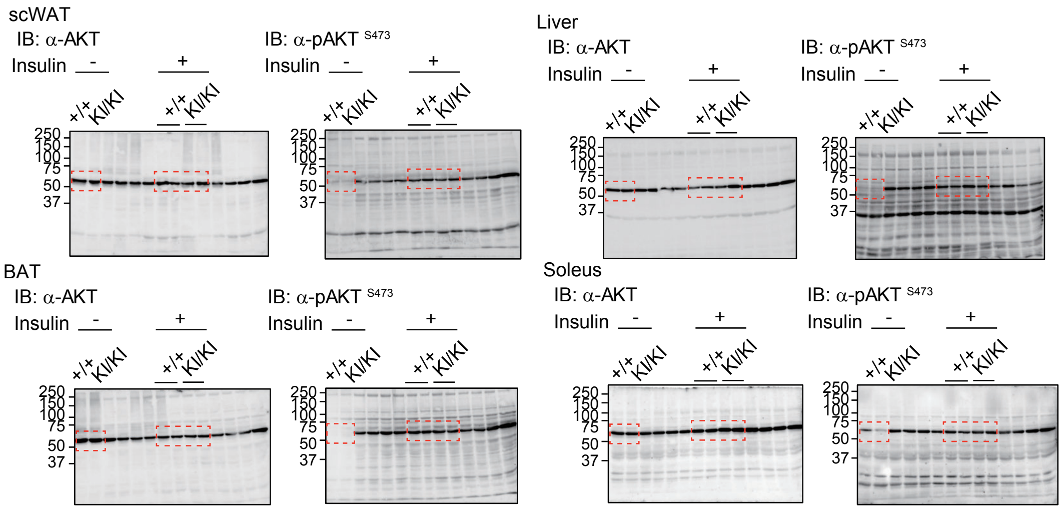

Supplementary Figure 8 Continued

Supplementary Table 1 Details of the age and sex of mice

Mouse Strain	Sex	Age (weeks at starting point of the treatment)	Figure
C57BL/6N	Male	6	Figure 1a,b
	Male	16	Figure 1c
	Male	8	Figure 1e
	Male	6	Figure 1f
	Male	6-10	Figure 1h
WT and Jmjd1a-S265A$^{KI/KI}$	Male	8-9	Figure 1j
	Male	7-8	Figure 1k
	Female	16-17	Figures 2a,b
	Male	22	Figure 2c
	Female	8-9	Figure 2d
	Male	7	Figure 2e
	Male	5	Figures 7a-d and Supplementary Figure 7a
	Male	9	Supplementary Figure 1k
	Male	44	Supplementary Figure 1n,p,q
	Male	8-9	Supplementary Figure 1o
	Male	7-9	Supplementary Figure 7b
WT (+/+) *and Jmjd1a*-null (-/-)	Female	6-11	Figure 5d
	Male	13	Supplementary Figures 1b (*WT*) and 2

Supplementary Table 2 Antibodies

Antibody		Source	Catalog No /Clone No	Dilutions or concentrations
anti-mouse JMJD1A	Monoclonal	Our laboratory	IgG-F0618	5 μg mL^{-1} for IB, 10 μg mL^{-1} for IP
anti-mouse JMJD1A	Monoclonal	Our laboratory	IgG-F0231	25 μg mL^{-1} for ChIP (together with 25 μg mL^{-1} of IgG-F0618)
anti-human JMJD1A	Monoclonal	Our laboratory	IgG-F1628	5 μg mL^{-1} for IB
anti-P-JMJD1A (pS265)	Polyclonal	Our laboratory	#11890-2	1:1000 for IB
anti-PPARγ	Monoclonal	Our laboratory	IgG-A3409	0.5 μg mL^{-1} for IB
anti-PPARγ	Monoclonal	Santa Cruz	sc-7273 (E-8)	4 μg mL^{-1} for ChIP (together with 4 μg mL^{-1} of IgG-A3409)
anti-PRDM16	Monoclonal	Our laboratory	IgG-F1411	5 μg mL^{-1} for IB (used in Fig. 4f (input))
anti-PRDM16	Monoclonal	Our laboratory	IgG-F1430	10 μg mL^{-1} for IP (used in Fig. 4g)
anti-PRDM16	Polyclonal	R&D Systems	AF6295	1 μg mL^{-1} for IB (used in Fig. 3c and 4f (IP ppt.) and Fig. 4g, Supplementary Fig. 4c-e)
anti-UCP1	Polyclonal	Abcam	ab10983	1:1000 for IHC
anti-UCP1	Monoclonal	R&D Systems	MAB6158	1 μg mL^{-1} for IB
anti-PGC1α	Polyclonal	Novus	1-04676	1:1000 for IB
anti-Total OXPHOS	Monoclonal	Abcam	ab110413	1.5 μg mL^{-1} for IB
anti-β-actin	Monoclonal	Sigma	A5441	1:5000 for IB
anti-V5	Monoclonal	Invitrogen	R960-25	1 μg mL^{-1} for IB, 10 μg mL^{-1} for IP
anti-H3K9me2	Monoclonal	Dr. Kimura	IgG-6D11	10 μg mL^{-1} for ChIP 2 μg mL^{-1} for IB
anti-Histone H3	Polyclonal	Abcam	ab-1791	0.05 μg mL^{-1} for IB
anti-AKT	Monoclonal	Cell Signaling	#4691	1:1000 for IB
anti-pAKT ^{S473}	Monoclonal	Cell Signaling	#4058	1:1000 for IB

Supplementary Table 3 ChIP-qPCR primers

**ChIP-qPCR primers**			
Gene	Sequence		Amplified regions
	Forward Primer	Reverse Primer	
Actb	5'-TGAGGTACTAGCCACGAGAGAG-3'	5'-ACACCCGCCACCAGGTAAGCA-3'	*Actb* (Intron 1)
Ppib	5'-CTCACCCCAACTAGTCTAATCC-3'	5'-GTGACACACAGTGACTAACTTCC-3'	*Ppib* (Intron 3)
Ucp1	5'-GCAACCCTCTCCCATCAGTG-3'	5'-GCCTAACACCGTGCTTCTCA-3'	*Ucp1* (-13 kb)
	5'-TGCAACCCCTCACCTTTTAC-3'	5'-CTCCTTCCATCATCCCTTCA-3'	*Ucp1* (-4.8 kb)
	5'-TCACCCTTGACCACACTGAA-3'	5'-GTGAGGCTGATATCCCCAGA-3'	*Ucp1* (-2.5 kb)
	5'-TGCCAAGTCCCACTAGCAG-3'	5'-ACCCGTTAAGCCCAGATTG-3'	*Ucp1* (TSS)
Ppara	5'-TGGCCGGGAGGAACTG-3’	5'-GGCAGGGACAATCTCTTTGTG-3'	*Ppara* (-10 kb)
	5'-GGCAGTCCCTTCACCTAACC-3'	5'-TCCTCGATGCCCATTTAGTG-3'	*Ppara* (TSS)
Cidea	5'-CACCGCTTCACTTTGTCCTTT-3'	5'-GAGCACCCGGTTTGACAGT-3'	*Cidea* (-13.5 kb)
	5'-CACGCACACCTGCTTCTCTA-3'	5'-GATGTTGGTGGCTCTTGTCA-3'	*Cidea* (TSS)

Supplementary Table 4 RT- qPCR primers

**RT- qPCR primers**		
Gene	Sequence	
	Forward Primer	Reverse Primer
Ppib #1	5'-GGAGATGGCACAGGAGGAA-3'	5'-GCCCGTAGTGCTTCAGCTT-3'
Ppib #2	5'-GCATACGGGTCCTGGCATCTTGT-3'	5'-ATGGTGATCTTCTTGCTGGTCTT-3'
Nd1	5'-GTTGGTCCATACGGCATTTT-3'	5'-TGGGTGTGGTATTGGTAGGG-3'
Nd2	5'-GCCTGGAATTCAGCCTACTAGC-3'	5'-GGCTGTTGCTTGTGTGACGA-3'
Nd4	5'-CGCCTACTCCTCAGTTAGCCA-3'	5'-TGATGTGAGGCCATGTGCGA-3'
Cytb	5'-CCTTCATGTCGGACGAGGCTT-3'	5'-TGCTGTGGCTATGACTGCGAA-3'
Cox1	5'-TAGCCCATGCAGGAGCATCA-3'	5'-TGGCTGGGGGTTTCATGTTGA-3'
Cox2	5'-ACCTGGTGAACTACGACTGCT-3'	5'-CCTAGGGAGGGGACTGCTCA-3'
Cox3	5'-CTTCACCATCCTCCAAGCTTCA-3'	5'-AGTCCATGGAATCCAGTAGCCAT-3'
Atp6	5'-TGGCATTAGCAGTCCGGCTT-3'	5'-ATGGTAGCTGTTGGTGGGCT-3'
Atp8	5'-TTCCCACTGGCACCTTCACC-3'	5'-TGTTGGGGTAATGAATGAGGCAA-3'
Ucp1	5'-AAGCTGTGCGATGTCCATGT-3'	5'-AAGCCACAAACCCTTTGAAAA-3'
Cidea	5'-GGTTCAAGGCCGTGTTAAGG-3'	5'-CGTCATCTGTGCAGCATAGG-3'
Pgc1a	5'-AACCACACCCACAGGATCAGA-3'	5'-TCTTCGCTTTATTGCTCCATGA-3'
Prdm16	5'-GCACGGTGAAGCCATTCATATG-3'	5'-TCGGCGTGCATCCGCTTGTG-3'
Dio2	5'-GTCCGCAAATGACCCCTTT-3'	5'-CCCACCCACTCTCTGACTTTC-3'
Cpt1b	5'-GCTGCCGTGGGACATTC-3'	5'-CTTGGCTACTTGGTACGAGTTCTC-3'
Adrb1	5'-GCTGGGAGTACGGCTCCTT-3'	5'-GCCGTCACACACAGCACAT-3'
Adrb3	5'-TCCTTCTACCTTCCCCTCCTT-3'	5'-CGGCTTAGCCACAACGAACAC-3'
Pparg	5'-CAAGAATACCAAAGTGCGATCAA-3'	5'-GAGCTGGGTCTTTTCAGAATAATAAG-3'
Adipoq	5'-CAGTGGATCTGACGACACCAA-3'	5'-GAACAGGAGAGCTTGCAACAGT-3'
Cox8b	5'-GCTGGCTGGACTCTGTCATT-3'	5'-GTACCAGGGCCTGCATAGTG-3'
Pgc1b	5'-GAGGGCTCCGGCACTTC-3'-3'	5'-CGTACTTGCTTTTCCCAGATGA-3'
Ppara	5'-ACAAGGCCTCAGGGTACCA-3'	5'-GCCGAAAGAAGCCCTTACAG-3'
Elovl3	5'-TTCTCACGCGGGTTAAAAATG-3'	5'-GGGCCTTAAGTCCTGAAACGT-3'
Bmp8b	5'-CACTTCCGCCGTGGAGC-3'	5'-GTGGGCTAAGACCCATCCTG-3'
Fabp4	5'-AGTGAAAACTTCGATGATTACATGAA-3'	5'-GCCTGCCACTTTCCTTGTG-3'
Nrf1	5'-TGCTTCAGAACTGCCAACCA-3'	5'-GGTCATTTCACCGCCCTGTA-3'
Irf4	5'-AGCTGCAAGTGTTTGCTCAC-3'	5'-GTCTGGCTAGCAGAGGTTCC-3'
Tfam	5'-CCGAAGTGTTTTTCCAGCAT-3'	5'-GGCTGCAATTTTCCTAACCA-3'
Fgf21	5'-CCTCTAGGTTTCTTTGCCAACAG-3'	5'-AAGCTGCAGGCCTCAGGAT-3'
